# ReDD-COFFEE under the Lens: Revealing Adsorption and Separation Performances of Hypothetical COFs Using Molecular Simulations and Machine Learning

**DOI:** 10.1021/acs.iecr.5c04806

**Published:** 2026-02-15

**Authors:** Hilal Ozyurt, Gokhan Onder Aksu, Hasan Can Gulbalkan, Seda Keskin

**Affiliations:** Department of Chemical and Biological Engineering, Koc University, Rumelifeneri Yolu, Sariyer, 34450 Istanbul, Turkey

## Abstract

In this work, we performed a high-throughput computational screening approach combining Grand Canonical Monte Carlo (GCMC) simulations and machine learning (ML) to unlock the potential of the ReDD-COFFEE (Ready-to-use and Diverse Database of Covalent Organic Frameworks with Force field-based Energy Evaluation) database for gas adsorption and separation applications. Molecular simulations were first employed to assess CO_2_, CH_4_, H_2_, N_2_ and O_2_ uptakes of acylhydrazone-, azine-, and triazine-based hypothetical COFs (hypoCOFs). These data were then leveraged to train ML models capable of predicting adsorption properties for nearly 25000 different types of materials. Adsorption selectivities of ReDD-hypoCOFs were computed for six important gas separations: CO_2_/CH_4_, CO_2_/H_2_, CO_2_/N_2_, CH_4_/H_2_, CH_4_/N_2_, and O_2_/N_2_. Structure-performance analyses performed using molecular fingerprinting on top-selective materials demonstrated that nitrogen enriched aromatic rings and fluorinated linkers in addition to narrow pores (<10 Å) and low porosities (<0.7) collectively strengthen the CO_2_ affinity of ReDD-hypoCOFs.

## Graphical abstract

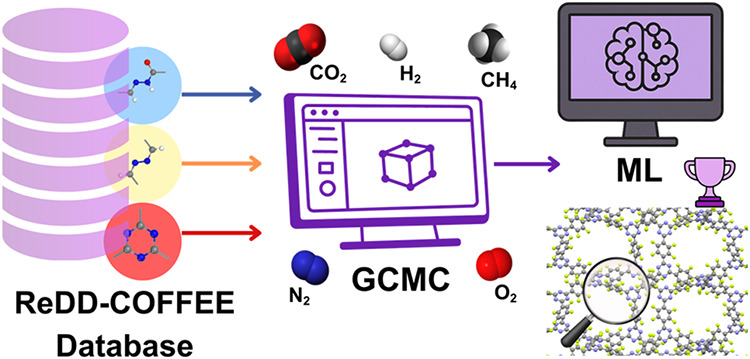

## 1 Introduction

Covalent organic frameworks (COFs) are an emerging family of porous solids with permanent porosities, large surface areas, and tunable pore sizes.^1–3^ They are composed of covalent bonds, which offer high structural stabilities and broad functional tunabilities.^4^ Thanks to these features, COFs have been widely studied for gas adsorption and separation applications as adsorbents and as membranes.^5–9^ The number of both experimentally synthesized and computationally generated (hypothetical) COFs has been growing rapidly. Due to the vast number of materials that need to be evaluated for different types of gas adsorption applications, molecular simulations have been widely used to explore this large material space.

Experimentally synthesized COF structures are consolidated in the Computation-Ready Experimental (CoRE) COF database, which consists of 1242 different types of COFs.^10^ To date, this database has been screened using molecular simulations for several adsorption-based gas separation applications. For example, 187 CoRE COFs were evaluated for noble gas separations using Grand Canonical Monte Carlo (GCMC) simulations for pressure-swing adsorption (PSA) and vacuum-swing adsorption (VSA) processes.^10^ Results revealed that COFs exhibit high Kr/Ar, Xe/Kr, Rn/Xe selectivities and high Kr and Xe working capacities. The same number of CoRE COFs were also examined for iodine and methyl iodide capture by GCMC simulations and results showed that three-dimensional COFs have higher uptakes compared to two-dimensional ones.^11^ Our group screened 295 CoRE COFs for CO_2_/N_2_:15/85 separation^12^ and 288 CoRE COFs for CO_2_/H_2_:15/85 separation^13^ using GCMC simulations at 0.1, 1, and 10 bar, 298 K. COF adsorbents were found to have high CO_2_/N_2_ selectivities in between 1–105 (1–66), and high CO_2_/H_2_ selectivities in between 2.4–625 (2.4–428) at 1 (10) bar.

Beyond the synthesized COFs, a hypothetical COF database, which consists of 69840 computer-generated but not yet synthesized structures, was introduced.^14^ This database was first evaluated for CH_4_ storage at 5.8 and 65 bar, 298 K by using GCMC simulations and results revealed that ∼300 hypoCOFs exhibit high CH_4_ deliverable capacities outperforming zeolites.^14^ The same database was also screened for the separation of CO_2_/N_2_:15/85 mixture for pressure–temperature swing adsorption (PTSA) process by GCMC simulations, and results showed that hypoCOFs can outperform synthesized COFs by achieving high CO_2_ working capacities (0.05 kg CO_2_/kg adsorbent).^15^ This hypoCOF database was also examined for adsorption-based separation of CO_2_/H_2_:15/85 mixture at 1 (10) bar, 298 K and hypoCOFs were reported to exhibit CO_2_/H_2_ selectivities up to 954 (742), outperforming the selectivities of synthesized COFs and zeolites. 3184 different hypoCOFs were studied by molecular simulations for H_2_S and CO_2_ capture from a six-component natural gas mixture, CH_4_/C_2_H_6_/CO_2_/C_3_H_8_/H_2_S/H_2_O, at 1 bar, 298 K and results showed that the top hypoCOFs have H_2_S + CO_2_ selectivities up to 7, comparable to those of carbon nanotubes and zeolites.^16^

As the number of materials in the COF spectrum increases very rapidly, performing molecular simulations for every single material and a large variety of gas molecules is simply impractical.^17^ Machine learning (ML) has been very recently combined with molecular simulations to evaluate thousands of materials and to reveal the hidden structure-performance relations. ML models are generally trained on the results of molecular simulations performed for a representative subset of COFs and then utilized to predict the desired results for the remaining COFs. In these ML models, structural and/or chemical features of COFs are generally used as input data, while target data is set as gas adsorption properties. In this way, the computational cost of molecular simulations that would require significant computational resources and time for evaluating thousands of materials can be largely bypassed by the ML predictions that can be obtained within seconds once the models are accurately trained.^18^

For example, CH_4_ adsorption capacities of 69839 hypoCOFs at 65 bar, 298 K were predicted by developing simple regression models trained with structural (pore size, surface area, density, void fraction) and chemical features (atom types) of COFs.^19^ The same set of materials was also evaluated for CH_4_ deliverable capacities (difference between CH_4_ uptakes at 65 and 5.8 bar, 298 K) by an active learning algorithm and the top-performing materials were identified.^20^ ML models were also developed to estimate CO_2_, CH_4_, H_2_, N_2_, and O_2_ uptakes of CoRE COFs and hypoCOFs at various pressures of 0.1, 1, 5, 10 bar, 298 K and results showed that hypoCOFs have higher uptakes and selectivities compared to synthesized COFs and zeolites.^21^ ML models were also used to evaluate hypoCOFs and CoRE COFs for the separation of equimolar CH_4_/H_2_,^22^ CO_2_/CH_4_,^23^ and C_3_H_8_/C_3_H_6_:15/85 mixtures^24^ and many hypoCOFs outperformed both synthesized COFs and zeolites by achieving higher selectivities.

By using the broadest diversity of linkage types and topological nets, a new hypoCOF database named as ReDD-COFFEE (Ready-to-use and Diverse Database of Covalent Organic Frameworks with Force field-based Energy Evaluation) has been recently introduced.^25^ This database comprises 268687 hypothetical COFs, which were constructed with different combinations of 279 secondary building units (SBUs), 1116 topologies, and 14 linkage types, which are the types of bonds between SBUs in each structure. These linkages are used to categorize ReDD-hypoCOFs into “chemical families” such as acylhydrazone, azine, and triazine. The ReDD-COFFEE database contains a wider variety of structures than CoRE COFs and Smit’s hypoCOF database as demonstrated through a comparative analysis of the structural and chemical properties.^25^ These structures were shown to achieve high thermal conductivities and mechanical stabilities.^26^ This database has been only studied for two different gas adsorption applications to date: The first study focused on evaluating CH_4_ deliverable capacities of ReDD-hypoCOFs using GCMC simulations at 5.8 and 65 bar, 298 K. Results showed that most of the ReDD-hypoCOFs (70.6%) achieve the ARPA-E (Advanced Research Projects Agency-Energy) target of 0.5 g/g for gravimetric deliverable capacity.^25^ Recently, the database was examined for CO_2_/N_2_:15/85 mixture separation by ML models using structural descriptors (pore size, surface area, pore volume) and chemical features (RACs) as input data, and ideal CO_2_/N_2_ selectivities and CO_2_ working capacities as target data. Results showed that imide, ketoenamine, and acylhydrazone families of ReDD-hypoCOFs can achieve high CO_2_/N_2_ selectivities up to 215 at 10 bar, 298 K.^27^

As this literature summary highlights, the potential of ReDD-hypoCOFs for industrially relevant gas separations remains unexplored. To address this gap, we combined molecular simulations with ML models to evaluate the gas adsorption capacities of nearly 25000 acylhydrazone, azine, and triazine ReDD-hypoCOFs. We first performed GCMC simulations on a representative subset of ReDD-hypoCOFs to determine their CO_2_, CH_4_, H_2_, N_2_, and O_2_ uptakes at 1 bar and 298 K. These results were then compared with the predictions obtained from the ML models originally developed for CoRE COFs in our previous work,^21^ allowing us to identify the hypothetical COF families where the ML models performed well and others where they required refinement. For the latter, we developed new ML models using structural, chemical, and energy-based features of triazine-based ReDD-hypoCOFs to assess their gas adsorption properties. Using the ML-predicted data, adsorption selectivities of 8366 acylhydrazone, 13367 azine, and 2359 triazine-typed ReDD-hypoCOFs were computed for six industrially important separations; CO_2_/CH_4_, CO_2_/H_2_, CO_2_/N_2_, CH_4_/H_2_, CH_4_/N_2_, and O_2_/N_2_. The separation performance of ReDD-hypoCOFs was benchmarked against CoRE COFs and previously reported Smit’s hypoCOFs. The top-performing ReDD-hypoCOFs offering the highest selectivity for each gas separation were identified and their structural and chemical features such as pore sizes, linkers, and linkage types were examined in detail. Our results will be useful for unlocking the gas adsorption and separation potentials of acylhydrazone, azine, and triazine-based hypothetical COFs and guiding the rational design of new COFs for high-performance adsorption-based gas separation applications.

## 2 Computational Details

Figure 1 summarizes our computational workflow which integrates molecular simulations and ML to evaluate gas adsorption and separation properties of ReDD-hypoCOFs.

**Figure 1:**
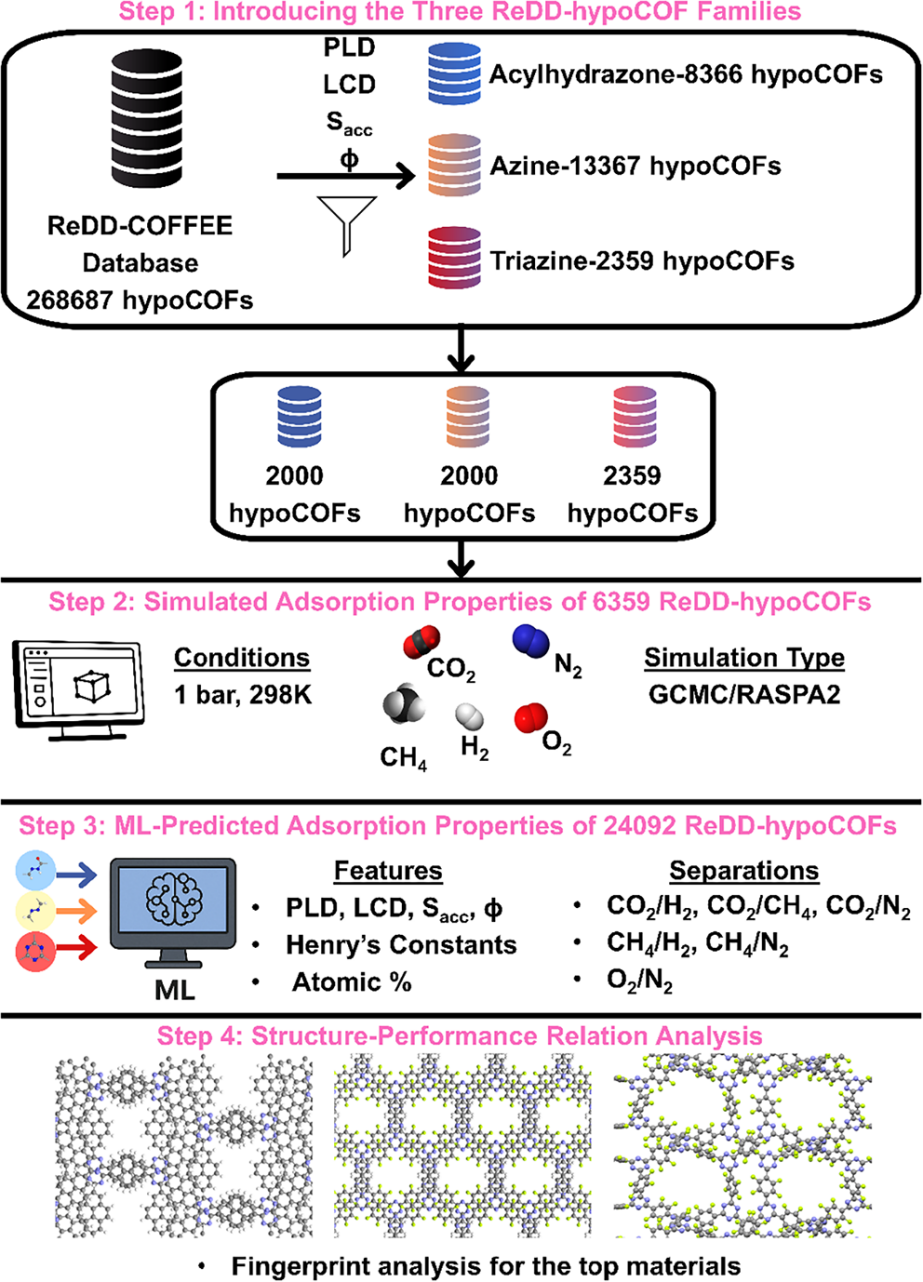
Overview of the computational workflow employed in this study: Step 1 introduces the three ReDD-hypoCOF families; acylhydrazone, azine, and triazine, selected from the ReDD-COFFEE database and their refined subsets designated for direct molecular simulations. Step 2 involves molecular simulations performed to compute the adsorption properties of CO_2_, CH_4_, H_2_, N_2_, and O_2_ in selected ReDD-hypoCOFs. Step 3 uses ML models trained on simulation data to predict adsorption properties for 24092 ReDD-hypoCOFs and to evaluate their CO_2_/H_2_, CH_4_/H_2_, CO_2_/N_2_, CO_2_/CH_4_, CH_4_/N_2_, and O_2_/N_2_ separation performances. PLD (Å), LCD (Å), surface area (m^2^/g), porosity (ϕ), Henry’s constants (mol/kg/Pa), atomic percentages (C, H, O, N, Halogen, Metalloid, and Ametal%) were used as input data. Step 4 identifies the top-performing ReDD-hypoCOFs and provides their molecular fingerprint analysis.

Our goal was to focus on representative chemical families for detailed analysis, enabling a meaningful comparison between molecular simulation results and ML predictions, rather than exhaustively screening all linkage types in the ReDD-COFFEE database. Acylhydrazone (R-C­(=O)­N­(H)­N=C-R) and azine (R-C=N–N=C-R) linkages were chosen to represent ReDD-COFFEE database, particularly because they are very rare or nonexistent in CoRE COF and other hypoCOF databases. Triazine (C_3_N_3_) differs from the other two families because of its aromatic ring structure, which allows us to assess how linkage chemistry influences gas adsorption and separation performance of COFs and to benchmark the transferability limits of ML models across chemically distinct linkage types exist in COFs.

### 2.1 Molecular Simulations

Our study concentrates on the ReDD-hypoCOF database^25^ encompassing 268687 different types of hypoCOFs. This database was classified according to the bond types between the linkers for each ReDD-hypoCOF. We focused on acylhydrazone, azine, and triazine ReDD-hypoCOFs, which comprise 39412, 55306, and 2641 structures, respectively. We calculated their structural properties such as pore limiting diameter (PLD), largest cavity diameter (LCD), accessible surface area (*S*_acc_), porosity (ϕ), and pore volume (PV), using the Zeo++ software (version 0.3).^28^ We eliminated materials with PLDs smaller than 3.8 Å and *S*_acc_ values of zero to ensure that all considered gases could be adsorbed within the pores.

Figure S1 shows that all acylhydrazone, azine, and triazine ReDD-hypoCOFs exhibit very high PLDs (0.5–333.9 Å), LCDs (1.4–337.6 Å), *S*_acc_ (0–10823 m^2^/g), and porosities (0.11–0.99). They have larger pores compared to CoRE COF and Smit’s hypoCOF structures (0.4–93 Å) and slightly higher surface areas (0–10260 m^2^/g). For using the ML models that we previously developed for CoRE COFs and making a fair comparison between ReDD-hypoCOFs and the other two COF databases in the next steps of our study, we further refined those families to retain materials having comparable structural properties to CoRE COFs and Smit’s hypoCOFs. For this refinement, we selected ReDD-hypoCOFs that have PLD, LCD, porosity, and surface area ranges of 4.2–92.2 Å, 5.5–92.5 Å, 0.42–0.97, and 385–9270 m^2^/g, respectively. As a result, a total of 8366 acylhydrazone, 13367 azine, and 2359 triazine ReDD-hypoCOFs were considered in this work. To reduce the computational cost of the subsequent molecular simulations, we randomly selected 2000 acylhydrazone and 2000 azine ReDD-hypoCOFs and included all triazine ReDD-hypoCOFs because 2359 structures are affordable for direct GCMC simulations. In our previous work,^21^ 1060 CoRE COFs and 6872 Smit hypoCOFs were studied using molecular simulations. We performed molecular simulations for a comparable number of hypoCOFs in the present study to ensure that the ReDD-COFFEE database is similarly well represented. Figure 2 shows the structural features for the refined set of acylhydrazone, azine, and triazine ReDD-hypoCOFs in comparison with CoRE COFs and Smit’s hypoCOFs. Figures S2 and S3 confirm that distributions of structural and energetic features in the randomly selected ReDD-hypoCOFs accurately represent those of the refined sets. Details on the number of structures that we studied for each family were summarized in Table S1.

**Figure 2:**
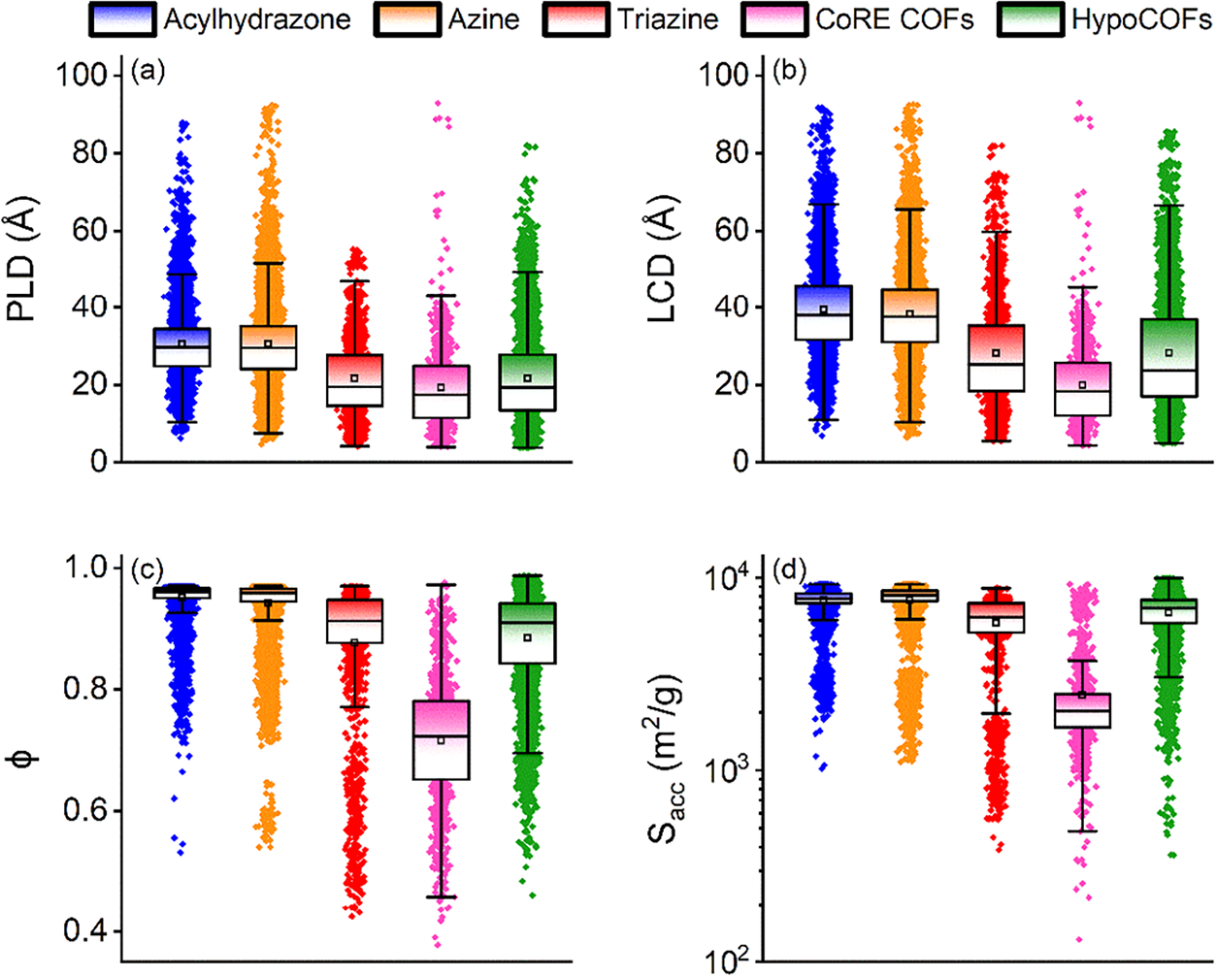
Computed structural features of 8366 acylhydrazone, 13367 azine, and 2359 triazine ReDD-hypoCOFs: (a) pore limiting diameter, (b) the largest cavity diameter, (c) porosity, (d) surface area. Data for 1060 CoRE COFs and 6872 Smit’s hypoCOFs are given for comparison.

We used the RASPA^29^ simulation software to compute CO_2_, CH_4_, H_2_, N_2_, and O_2_ uptakes (N_i_) of 2000 acylhydrazone, 2000 azine, and 2359 triazine ReDD-hypoCOFs at 1 bar, 298 K by performing GCMC simulations. The intermolecular interactions in these simulations were described using the Lennard-Jones (12–6) potential for gas–gas and gas-framework interactions, while the DREIDING force field was used for the COFs.^30^ For CO_2_, N_2_, and O_2_ adsorption simulations, we assigned the partial point charges to materials using the charge equilibration method (Qeq)^31,32^ integrated into the RASPA^29^ simulation software. CO_2_ was represented as a rigid three-site linear model, with a C–O bond length of 1.16 Å and partial charges positioned at the center of each site.^33^ CH_4_ and H_2_ were treated as single-site molecules.^34,35^ N_2_ and O_2_ were described as rigid three-site models, where the atoms occupied the terminal sites and the center of mass served as the third site.^36,37^ The simulation box length was chosen to be at least 28 Å, ensuring compatibility with a cutoff distance of 14 Å. Electrostatic interactions between CO_2_, N_2_, O_2_ molecules and COFs were calculated using the Coulombic potential with the Ewald summation.^38^ GCMC simulations were carried out with 10000 cycles dedicated to system equilibration, followed by 20000 cycles to calculate the average properties. The Henry’s constants and heat of adsorption values for CO_2_, CH_4_, H_2_, N_2_, and O_2_ were also computed through the Widom insertion technique^39^ by using 50000 cycles. We note that the accuracy of our molecular simulations was previously validated by comparing the simulation results with the experimentally measured single-component CO_2_ adsorption^12^ in COF-5, COF-6, COF-8, and COF-10 up to 20 bar, 298 K and CH_4_ adsorption^40^ in COF-8, COF-10, COF-102, and COF-103 up to 30 bar, 298 K.

### 2.2 Machine Learning

The vast diversity of COF structures makes it impractical to perform direct molecular simulations for every material due to the high computational cost. To overcome this limitation, we previously developed ML models trained on the molecular simulation data of 1060 CoRE COFs and 563 Smit’s hypoCOFs for CO_2_, CH_4_, H_2_, N_2_, and O_2_ adsorption together with their structural, chemical, and energetic features.^21^ In these models, the feature set included four structural descriptors (PLD, LCD, *S*_acc_, and ϕ); seven chemical descriptors (percentages of carbon, hydrogen, nitrogen, oxygen, halogens, ametals, and metalloids in COFs) extracted from the crystallographic information files (CIFs) obtained from the corresponding COF databases; and an energy-based descriptor (Henry’s coefficients for the five gases). All these models were developed using Tree-based Pipeline Optimization Tool (TPOT) (version 0.12.2). The details of the previously developed models are available at the following GitHub link: https://github.com/gokhanonderaksu/COFSpace/tree/main

We used these ML models to predict CO_2_, CH_4_, H_2_, N_2_, and O_2_ uptakes of 2000 acylhydrazone, 2000 azine, and 2359 triazine ReDD-hypoCOFs at 1 bar, 298 K and analyzed their prediction accuracies by comparing with the simulated uptakes. We observed that our previous ML models made accurate gas adsorption predictions for acylhydrazone and azine families, but they failed to predict gas uptakes of triazine ReDD-hypoCOFs, as will be discussed in detail in the following section. Therefore, we trained new ML models for 2359 triazine ReDD-hypoCOFs using the same feature set (except percentage of ametals, which are all zero for these structures) as the input data and the simulation data for CO_2_, CH_4_, H_2_, N_2_, and O_2_ uptakes of triazine ReDD-hypoCOFs as the target data.

For training new ML models, TPOT^41^ (version 0.12.2) was utilized to identify the optimal models and to perform hyperparameter tuning. Regression models from the scikit-learn library^42^ were employed for selecting the best-performing models. To ensure a consistent distribution of features between the training and test sets, a stratified sampling method was applied, with 80% of the data used for training and the remaining 20% for testing. The performance of the new ML models was evaluated using multiple statistical metrics, including the coefficient of determination (*R* ^2^), mean absolute error (MAE), root-mean-square error (RMSE), and Spearman’s rank correlation coefficient (SRCC), as provided in Table S2. The optimized hyperparameters of our new ML models for triazine ReDD-hypoCOFs are presented in Table S3. Correlation matrices were also used to examine the interdependence in the feature set used in these new ML models, as shown in Figure S4. These new ML models are available in our Github repository, https://github.com/hilalozyurt/ReDD-hypoCOFs_ML together with the all structural, chemical, and energetic features of the triazine ReDD-hypoCOFs.

SHapley Additive ExPlanations (SHAP)^43^ analysis was applied to evaluate how each feature influenced the ML predictions, in both magnitude and direction. The SHAP values (positive or negative) were calculated as the difference between model’s predictions for the simulated gas uptakes and the average prediction for these simulated uptakes. Overall, CO_2_, CH_4_, H_2_, N_2_, and O_2_ gas uptakes of 8366 acylhydrazone and 13367 azine ReDD-hypoCOFs, a total of 21733 structures, were predicted by using our previous ML models, while the gas uptakes for 2359 triazine ReDD-hypoCOFs were estimated by using our newly developed ML models. As a result, 24092 ReDD-hypoCOFs belonging to these three families were examined and top-performing materials were efficiently identified. Based on the ratio of ML-predicted gas uptakes, the selectivities for CO_2_/CH_4_, CH_4_/H_2_, CO_2_/N_2_, CO_2_/H_2_, and O_2_/N_2_ separations were subsequently calculated and the best-performing ReDD-hypoCOF materials demonstrating the highest selectivities were identified. Then, these materials were analyzed based on their structural and chemical features with an in-depth molecular-level investigation assisted by molecular fingerprinting technique, MACCSKeys,^44^ to elucidate the critical structural and chemical features that are responsible for high selectivities.

## 3 Results and Discussion

Figure 3 shows the simulated CO_2_, CH_4_, H_2_, N_2_, and O_2_ uptakes of 2000 acylhydrazone, 2000 azine, and 2359 triazine ReDD-hypoCOFs at 1 bar. ReDD-hypoCOFs exhibit gas adsorption preferences with the following order: CO_2_> CH_4_> N_2_ ≈ O_2_> H_2_. Figure 3(a) shows that acylhydrazone, azine, triazine ReDD-hypoCOFs have CO_2_ uptakes in the ranges of 0.56–2.42, 0.39–3.51, and 0.37–6.95 mol/kg, respectively, at 1 bar and 298 K. There are 225 ReDD-hypoCOFs having high CO_2_ uptakes (>2 mol/kg), and 92% (207 out of 225) of these belong to triazine family, underlining the potential of triazine-based COFs for CO_2_ capture. Triazine ReDD-hypoCOFs also exhibit higher CH_4_ uptakes (0.21–1.65 mol/kg) than acylhydrazone family (0.36–1.04 mol/kg) and similar CH_4_ uptakes to azine family (0.27–1.48 mol/kg) as shown in Figure 3(b). Only 15 ReDD-hypoCOFs show high CH_4_ uptakes (>1.2 mol/kg), and 14 of these are triazine-based COFs. All ReDD-hypoCOF families achieve very similar O_2_ and N_2_ capacities: O_2_ uptakes of acylhydrazone, azine, and triazine ReDD-hypoCOFs were computed in between 0.16–0.89, 0.13–0.92, and 0.11–0.90 mol/kg, respectively, at 1 bar as shown in Figure 3(c). N_2_ adsorption of acylhydrazone, azine, and triazine ReDD-hypoCOFs span the ranges of 0.16–0.88, 0.14–0.92, and 0.10–0.89 mol/kg, respectively, as shown in Figure 3(d). In Figure 3(e), H_2_ uptakes of acylhydrazone, azine, and triazine ReDD-hypoCOFs were computed in between 0.03–0.73, 0.03–0.76, and 0.02–0.71 mol/kg, respectively, showing that all three families have similar weak adsorption affinity for H_2_.

**Figure 3:**
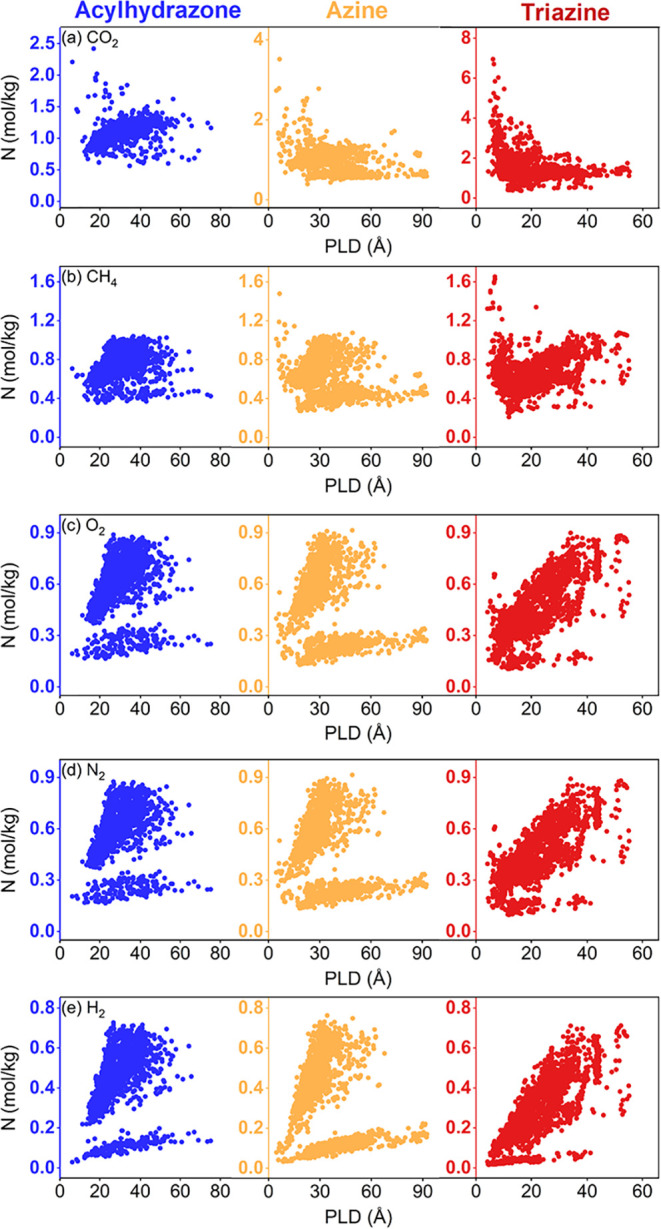
Simulated (a) CO_2_, (b) CH_4_, (c) O_2_, (d) N_2_, and (e) H_2_ uptakes for 2000 acylhydrazone, 2000 azine, and 2359 triazine ReDD-hypoCOFs at 1 bar, 298 K, plotted as a function of PLD.

Figure 4 shows adsorption selectivities of ReDD-hypoCOFs computed from the simulated gas uptakes obtained at 1 bar, 298 K. Selectivities that we previously reported^21^ for CoRE COFs and Smit’s hypoCOFs were provided for comparison. Selectivities of ReDD-hypoCOFs were computed in the range of 1.6–179.6, 1.4–43.4, 1.3–22.3, 1.2–10.4, 1.1–4.3, and 0.8–1.4 for CO_2_/H_2_, CH_4_/H_2_, CO_2_/N_2_, CO_2_/CH_4_, CH_4_/N_2_, and O_2_/N_2_ separations, respectively, showing that they are CO_2_ selective over other gases and CH_4_ selective over H_2_ and N_2_. CO_2_/CH_4_ selectivities of acylhydrazone, azine, and triazine families were computed as 1.2–5.0, 1.2–8.5, and 1.2–10.4, respectively, as given in Figure 4(a). Among 67 ReDD-hypoCOFs exhibiting CO_2_/CH_4_ selectivities >5, 79% (53 out of 67) of them are triazine ReDD-hypoCOFs, highlighting their strong potential for natural gas purification. ReDD-hypoCOFs have similar CO_2_/CH_4_ selectivities to Smit’s hypoCOFs (1.1–8.2) and CoRE COFs (1.2–17.5).

**Figure 4:**
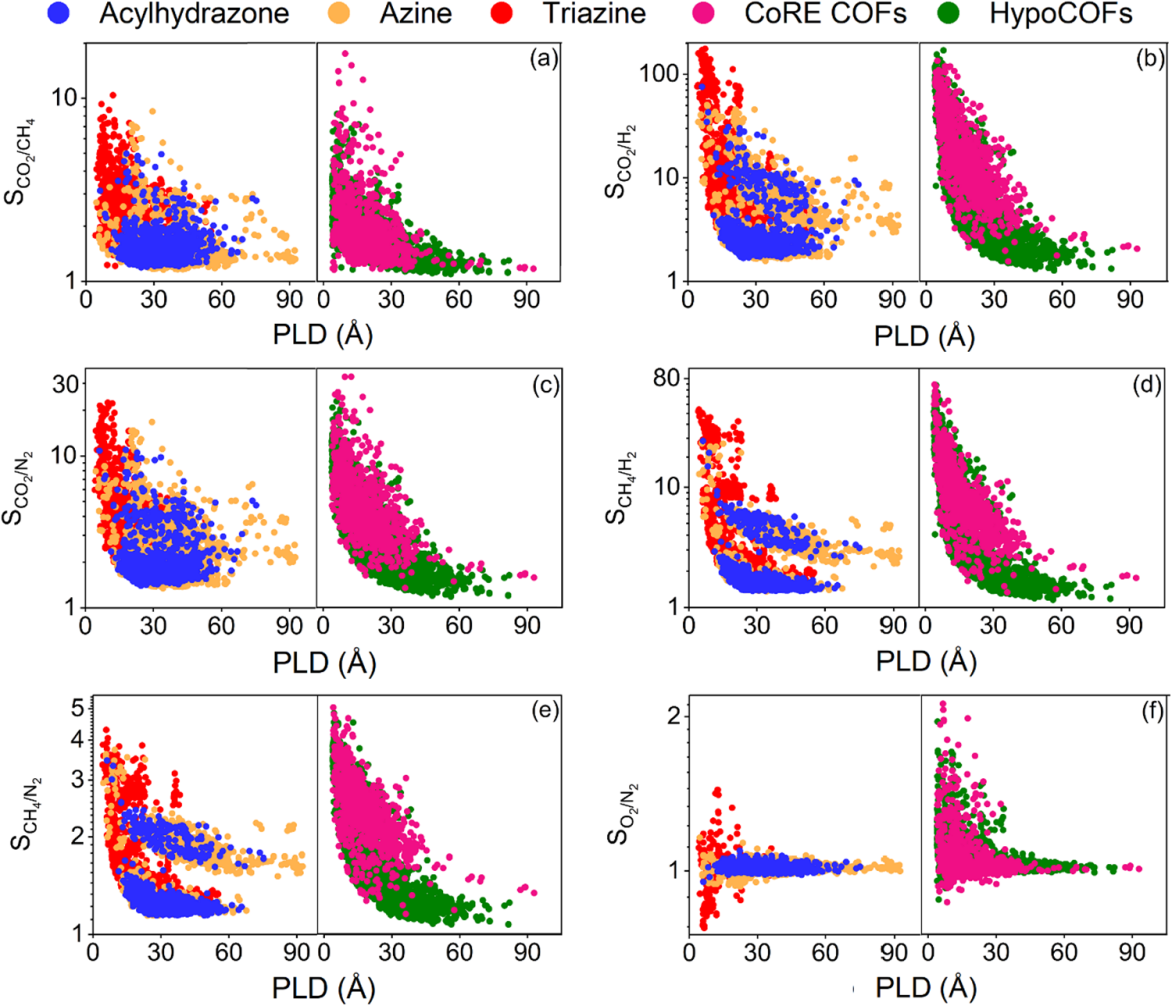
Simulated adsorption selectivities of 2000 acylhydrazone (blue), 2000 azine (orange), and 2359 triazine (red) ReDD-hypoCOFs for (a) CO_2_/CH_4_, (b) CO_2_/H_2_, (c) CO_2_/N_2_, (d) CH_4_/H_2_, (e) CH_4_/N_2_, and (f) O_2_/N_2_ separations at 1 bar, 298 K. Data for 6872 Smit’s hypoCOFs (green) and 1060 CoRE COFs (pink) were included for comparison.

In Figure 4(b), CO_2_/H_2_ selectivities of acylhydrazone, azine, and triazine ReDD-hypoCOFs are shown as 1.7–75.8, 1.6–50.4, and 1.8–179.6, respectively. 38 triazine ReDD-hypoCOFs exhibit selectivities exceeding 100, while there is no acylhydrazone or azine-based structure reaching for that benchmark. We identified 21 triazine ReDD-hypoCOFs achieving higher selectivities than the highest value computed for CoRE COFs (135), and a triazine ReDD-hypoCOF exhibiting higher selectivity than the highest value of Smit’s hypoCOFs (170) for CO_2_/H_2_ separation. Figure 4(c) presents CO_2_/N_2_ selectivities of acylhydrazone, azine, and triazine ReDD-hypoCOFs, calculated in the ranges of 1.4–10.9, 1.3–16.7, and 1.5–22.3, respectively. There are 30 ReDD-hypoCOFs having high CO_2_/N_2_ selectivities, >15, and 29 of these belong to triazine family. These high selectivities are comparable to those of Smit’s hypoCOFs (1.2–22.9) but lower than the range observed for CoRE COFs (1.3–33.2).

Triazine ReDD-hypoCOFs exhibit higher adsorption selectivities (1.5–43.4) compared to acylhydrazone (1.4–24.2) and azine (1.4–23.4) families for CH_4_/H_2_ separation, as shown in Figure 4(d). There are 138 ReDD-hypoCOFs exhibiting high CH_4_/H_2_ selectivities (>20), of which 94.2% (130 out of 138) belong to triazine ReDD-hypoCOFs. However, their selectivity range is lower compared to that of CoRE COFs (1.3–72.6) and Smit’s hypoCOFs (1.2–71.9) for CH_4_/H_2_ separation. In Figure 4(e), CH_4_/N_2_ selectivities of acylhydrazone, azine, and triazine ReDD-hypoCOFs were shown as 1.1–3.4, 1.1–3.7, and 1.2–4.3, respectively. These selectivities are similar to those observed for CoRE COFs (1.2–5.1) and Smit’s hypoCOFs (1.1–4.8). Finally, Figure 4(f) shows that O_2_/N_2_ selectivities of all ReDD-hypoCOFs are between 0.8 and 1.4, like the ones computed for CoRE COFs (0.8–2.1) and Smit’s hypoCOFs (0.9–1.9), showing that COFs can be O_2_ or N_2_ selective, but their preference for either component is weak. Overall, ReDD-hypoCOFs outperform Smit’s hypoCOFs and CoRE COFs in CO_2_/H_2_ separation, while exhibiting comparable performance for other gas separations.

The next step was to utilize our previous ML models, trained with the data of CoRE COFs, to predict CO_2_, CH_4_, H_2_, N_2_, and O_2_ uptakes of all acylhydrazone, azine, and triazine ReDD-hypoCOFs, including the ones for which gas uptakes have not been computed by molecular simulations. Before using these ML models for the entire ReDD-hypoCOF data set, we first tested their transferability to unseen subsets for which we have the simulation data. Figure 5 presents the comparison of ML-predicted and simulated CO_2_, CH_4_, H_2_, N_2_, and O_2_ uptakes for 2000 acylhydrazone and 2000 azine frameworks, which were not used in training or testing the ML models. As Figure 5(a,b) show our ML models achieve good accuracy for predicting CO_2_ and CH_4_ uptakes in acylhydrazone and azine ReDD-hypoCOFs, resulting in *R* ^2^ >0.8, MAE< 0.1 mol/kg, and SRCC >0.95. ML-predicted CO_2_ (CH_4_) uptakes of acylhydrazone and azine were estimated in the range of 0.58–2.26 (0.35–1.08) mol/kg and 0.39–3.55 (0.27–1.39) mol/kg, respectively, which are in a good agreement with those of simulated values, 0.56–2.42 (0.36–1.04) mol/kg and 0.39–3.51 (0.27–1.48) mol/kg. In Figure 5(c,d), our ML models estimated O_2_ (N_2_) uptakes of acylhydrazone and azine families in the ranges of 0.17–0.95 (0.17–0.91) mol/kg and 0.13–0.97 (0.14–0.92) mol/kg, respectively, closely matching simulated uptakes of both families, 0.16–0.89 (0.16–0.88) mol/kg and 0.13–0.92 (0.14–0.92) mol/kg. The statistical accuracy metrics of our ML models for these two gases were high with *R* ^2^ (>0.95) and SRCC (>0.95).

**Figure 5:**
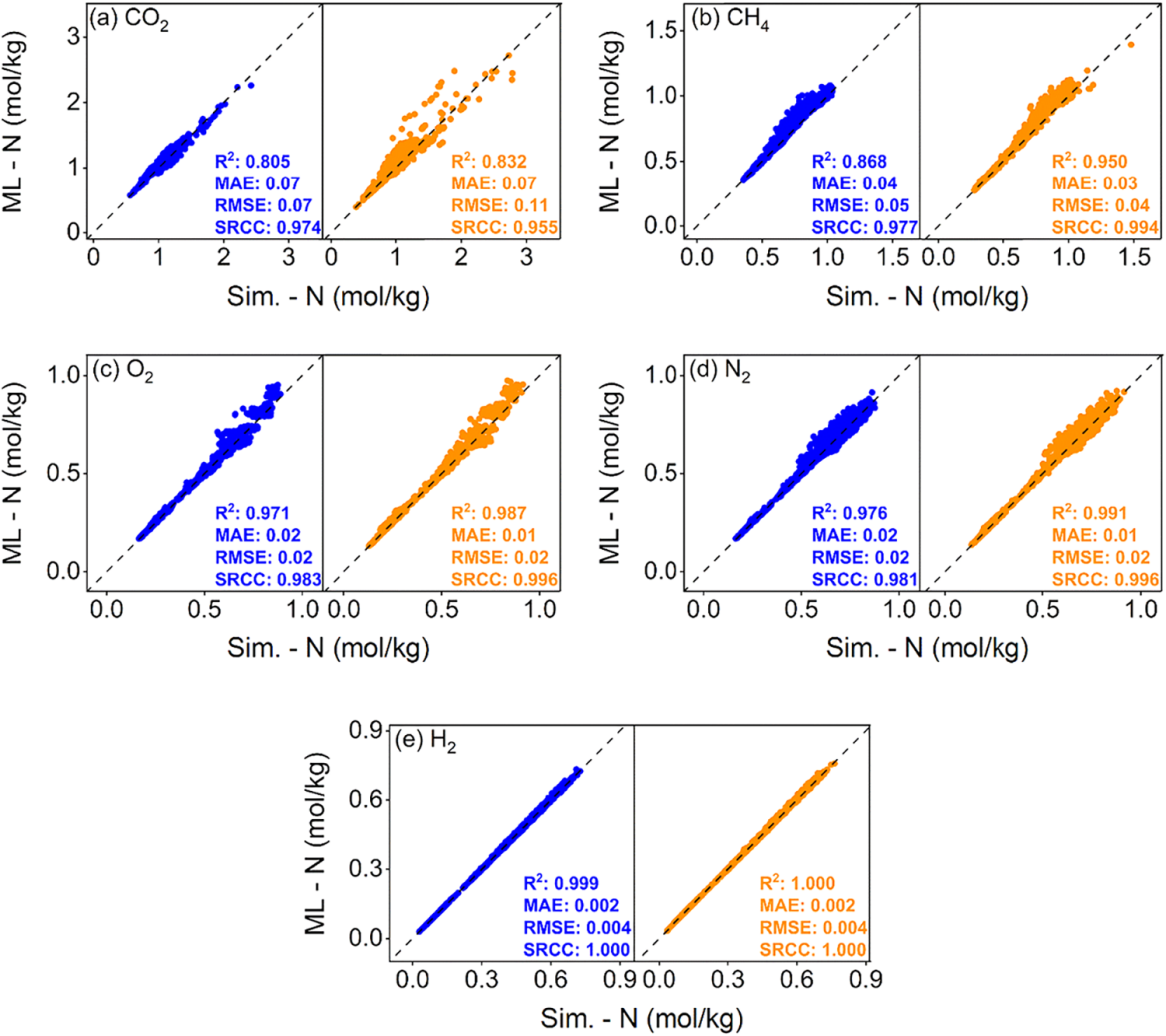
Comparison of simulated and ML-predicted gas uptakes for (a) CO_2_, (b) CH_4_, (c) O_2_, (d) N_2_, and (e) H_2_ for 2000 acylhydrazone (blue) and 2000 azine ReDD-hypoCOFs (orange) at 1 bar and 298 K.

H_2_ uptakes of acylhydrazone and azine ReDD-hypoCOFs were well predicted by ML models leading to very high *R* ^2^ and SRCC scores (∼1), as shown in Figure 5(e). ML-predicted H_2_ uptakes of acylhydrazone and azine ReDD-hypoCOFs change in between 0.03–0.73 and 0.03–0.76 mol/kg, respectively, as the simulated ones have the same ranges. The top 50 acylhydrazone and azine ReDD-hypoCOFs with the highest simulated H_2_ uptakes were all identified by our ML models’ predictions as well. Overall, we confirmed the transferability of ML models originally trained for CoRE COFs to acylhydrazone and azine ReDD-hypoCOF families for accurately predicting their CO_2_, CH_4_, H_2_, N_2_ and O_2_ gas uptakes at 1 bar, 298 K.

For triazine-based ReDD-hypoCOFs, Figure S5­(a–c) show that our ML models accurately predicted O_2_, N_2_, and H_2_ uptakes. ML-predicted gas uptakes (0.11–0.88, 0.1–0.87, 0.02–0.71 mol/kg, respectively) closely matched the simulated ones (0.11–0.89, 0.1–0.89, and 0.02–0.71 mol/kg, respectively). On the other hand, ML models overestimated CO_2_ and CH_4_ uptakes of triazine structures, 0.46–7.20 and 0.23–2.62 mol/kg, compared to simulation results of 0.37–6.95 and 0.21–1.65 mol/kg, as shown in Figure S5­(d,e). This overestimation led to very low *R* ^2^ (0.05 and 0.38) values for CO_2_ and CH_4_. The reason for such overpredictions was found to be related to the different correlations observed for triazine structures between CO_2_ and CH_4_ uptakes and their Henry’s constants, which are the most important features in our ML models. Figure S6 shows that Henry’s constants of CO_2_ and CH_4_ are highly correlated with their uptakes (the Pearson’s constants (r) of 0.61 and 0.91, respectively) for CoRE COFs, which were used in training our ML models. On the other hand, Pearson’s constants between uptakes and Henry’s constants of CO_2_ and CH_4_ for triazine ReDD-hypoCOFs were estimated as 0.24 and 0.72, showing much weaker correlation. Figure S6­(a) shows that for CoRE COFs high CO_2_ Henry’s constants are associated with high CO_2_ uptakes. For example, there are 205 COFs having high Henry’s constants of CO_2_ (>10^–4^ mol/kg/Pa) among training set of our models, and their simulated CO_2_ uptakes were reported up to 14 mol/kg. In Figure S6­(b), there are 66 triazine ReDD-hypoCOFs with high CO_2_ Henry’s constants and their simulated CO_2_ uptakes are in between 1–7 mol/kg, while ML predictions are 3.2–7.2 mol/kg. Similarly, Figure S6­(c) shows that there are 631 COFs having high CH_4_ Henry’s constants (>10^–5^ mol/kg/Pa), and they achieve high CH_4_ uptakes up to 7.6 mol/kg. Figure S6­(d) shows that 122 triazine ReDD-hypoCOFs with high Henry’s constants have simulated and ML-predicted CH_4_ uptakes in between 0.9–2.6 and 0.6–1.7 mol/kg, respectively. Overall, our previous ML models cannot capture and perform accurate CO_2_ and CH_4_ uptake predictions for triazine ReDD-hypoCOFs due to differences in underlying relationships with the Henry’s constants.

Therefore, the next step was to develop new ML models to reflect and capture the underlying relationships between gas uptakes and structural, chemical, and energy-based features of triazine ReDD-hypoCOFs. Figure 6 represents the analysis of our new ML models trained with the simulation data of 2359 triazine ReDD-hypoCOFs to predict their CO_2_, CH_4_, H_2_, N_2_ and O_2_ uptakes at 1 bar. Our new ML models accurately predict all gas uptakes of triazine-based ReDD-hypoCOFs, as high *R* ^2^ (>0.9), SRCC (>0.9) and low MAE (<0.01) values were achieved in the test sets.

**Figure 6:**
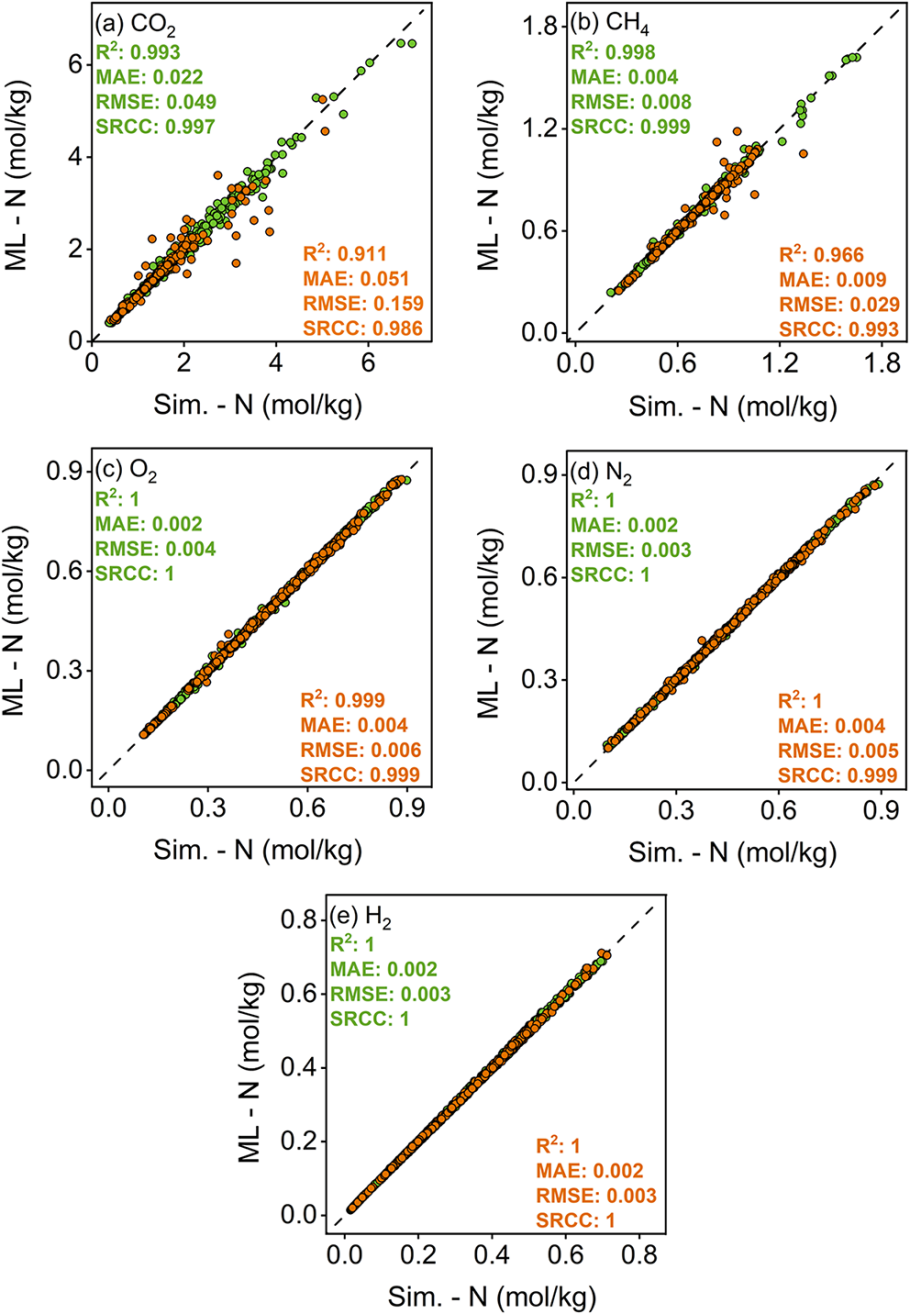
Comparison of ML-predicted (a) CO_2_, (b) CH_4_, (c) O_2_, (d) N_2_, and (e) H_2_ uptakes of 2359 triazine ReDD-hypoCOFs with their corresponding simulated uptakes at 1 bar. Green (orange) data points represent training (test) sets.

In Figure 6(a), ML-predicted CO_2_ uptakes were estimated in between 0.40–6.47 mol/kg, closely matching simulated ones, 0.37–6.95 mol/kg. Similarly, ML-predicted CH_4_ uptakes change in between 0.24–1.62 mol/kg, which is almost the same for simulated ones, 0.21–1.65, as shown in Figure 6(b). New ML models identified 43 (45) of the top 50 triazine structures with the highest CO_2_ (CH_4_) uptakes, accurately pinpointing promising materials, as this success was also reflected in very high SRCC scores. Figure 6(c–e) show that ML-predicted O_2_, N_2_ and H_2_ uptakes are 0.11–0.88, 0.10–0.87, and 0.02–0.71 mol/kg, which are in strong agreement with the simulated ones, 0.11–0.90, 0.10–0.89 and 0.02–0.71 mol/kg, respectively. As their SRCC scores are 1, all our corresponding ML models accurately pinpointed the top 50 triazine structures with the highest O_2_, N_2_, and H_2_ uptakes. Figure S7 shows that Henry’s constants of gases are the most dominant features of ML models. Their high (low) values are strongly associated with ML models’ high (low) predictions.

Finally, we used our newly constructed ML models for triazines and previous ML models for acylhydrazone and azine ReDD-hypoCOFs to assess the gas uptake properties of all three families. Figure 7 presents the comparison of ML-predicted gas uptakes for 8366 acylhydrazone, 13367 azine, and 2359 triazine ReDD-hypoCOFs at 1 bar and 298 K. Figure 7(a) shows that acylhydrazone, azine, and triazine ReDD-hypoCOFs have CO_2_ uptakes of 0.57–2.89, 0.39–3.56, and 0.41–6.47 mol/kg, respectively. A total of 272 ReDD-hypoCOFs exhibit CO_2_ uptakes greater than 2 mol/kg and 78.3% (213 of 272) of which belong to the triazine family. Figure 7(b) presents that triazine ReDD-hypoCOFs have higher CH_4_ uptakes (0.24–1.61 mol/kg) than acylhydrazones (0.33–1.17 mol/kg) but comparable to azines (0.27–1.71 mol/kg). Six triazine and one azine structure show CH_4_ uptakes greater than 1.5 mol/kg. Figure 7(c,d) shows O_2_ and N_2_ uptakes of acylhydrazone, azine, and triazine ReDD-hypoCOFs in the ranges of 0.15–1, 0.13–1.05, 0.11–0.88 mol/kg and 0.16–0.93, 0.13–0.96, 0.10–0.87 mol/kg, respectively. As shown in Figure 7(e), H_2_ uptakes of all families are low reaching to maximum of 0.75 mol/kg.

**Figure 7:**
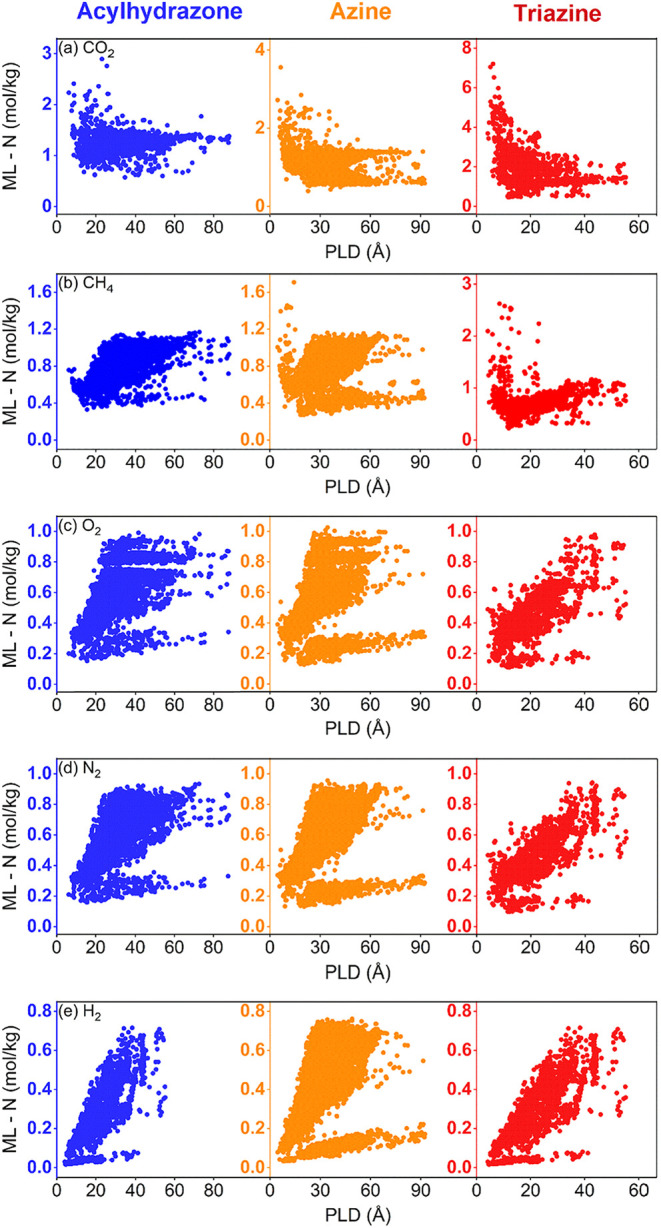
Comparison of ML-predicted (a) CO_2_, (b) CH_4_, (c) O_2_, (d) N_2_, and (e) H_2_ uptakes of 8366 acylhydrazone, 13367 azine, 2359 triazine ReDD-hypoCOFs, with respect to their pore sizes.

Using the ML-predicted gas uptakes, we computed selectivities of ReDD-hypoCOFs for various gas separations, as shown in Figure 8. Considering all 24092 frameworks together (8366 acylhydrazone, 13367 azine, 2359 triazine), ML-predicted CO_2_/CH_4_, CO_2_/H_2_, CO_2_/N_2_, CH_4_/H_2_, CH_4_/N_2_, and O_2_/N_2_ selectivities span 1.2–9.2, 1.8–189.5, 1.5–21.0, 1.5–48.7, 1.2–4.3, and 0.8–1.4, respectively. Figure 8(a) shows the ML-predicted CO_2_/CH_4_ selectivities of acylhydrazone, azine, and triazine ReDD-hypoCOFs, ranging between 1.2–8.0, 1.2–9.1, and 1.3–9.2 respectively. 52, 31, and 4 triazine, azine, and acylhydrazone structures achieved CO_2_/CH_4_ selectivities >5, respectively. Figure 8(b) presents the CO_2_/H_2_ selectivities, which change in between 1.8–76.3, 1.8–71.2, and 1.8–189.5 for acylhydrazone, azine, and triazine ReDD-hypoCOFs, respectively. Among these, 39 triazine structures exhibit CO_2_/H_2_ selectivities greater than 100, while no acylhydrazone or azine ReDD-hypoCOF reaches this level. CO_2_/N_2_ selectivities of acylhydrazone, azine, and triazine families are very similar in the ranges; 1.5–15, 1.5–20, and 1.5–21, respectively, as shown in Figure 8(c). Among them, 33 ReDD-hypoCOFs have CO_2_/N_2_ selectivities greater than 15, and 91% (30 out of 33) are triazine-based structures.

**Figure 8:**
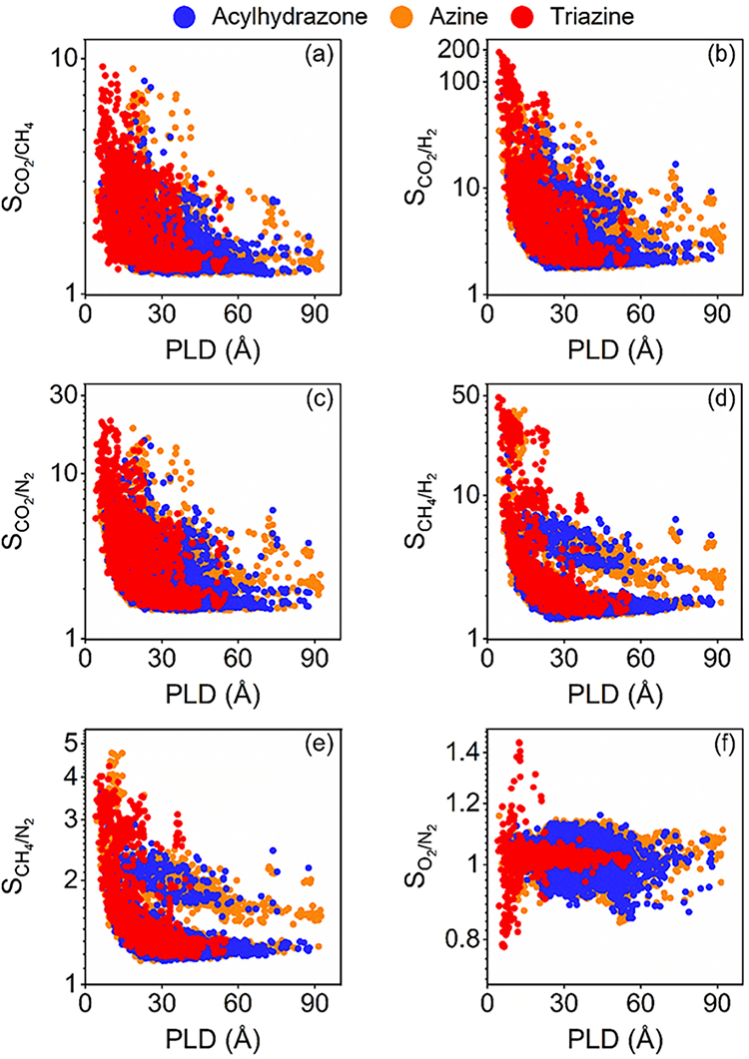
Comparison of ML-predicted (a) CO_2_/CH_4_, (b) CO_2_/H_2_, (c) CO_2_/N_2_, (d) CH_4_/H_2_, (e) CH_4_/N_2_, and (f) O_2_/N_2_ selectivities of 8366 acylhydrazone, 13367 azine and 2359 triazine ReDD-hypoCOFs, with respect to their pore sizes.

Figure 8(d) illustrates that CH_4_/H_2_ selectivities of acylhydrazone, azine, and triazine ReDD-hypoCOFs change in between 1.4–26, 1.4–40, and 1.5–49, respectively. Among them, 162 ReDD-hypoCOFs have CH_4_/H_2_ selectivities greater than 20, and 81% (131 out of 162) are triazine-based structures. Figure 8(e) shows low CH_4_/N_2_ selectivities of acylhydrazone, azine, and triazine families, between 1.2–3.6, 1.2–5, and 1.2–4.3, respectively. Similarly, Figure 8(f) displays O_2_/N_2_ selectivities of acylhydrazone, azine, and triazine ReDD-hypoCOFs, ranging between 0.9 and 1.2, 0.8–1.2, and 0.7–1.5, respectively, and demonstrating these materials’ nonselective behavior for this separation.

These results indicate that the three families that we studied as adsorbents have the most potential for CO_2_ separations (CO_2_/CH_4_, CO_2_/H_2_, CO_2_/N_2_) and CH_4_/H_2_ separation. Among the three families, triazine-based ReDD-hypoCOFs consistently populate the highest selectivity regime. This can be attributed to the Lewis basicity of the triazine nitrogen atoms, which facilitates interactions with the Lewis acidic CO_2_ molecules.^45^ Acylhydrazone (R-C­(=O)­N­(H)­N=C-R) and azine (R-C=N–N=C-R) families have extensive π-conjugation across the linkages, which leads to delocalized electron density and making them poor Lewis bases.^46^ In addition, triazine rings (C_3_N_3_) are electron deficient aromatic units, and they create strong quadrupole–quadrupole interactions with CO_2_ molecules.^47^

We ranked all ReDD-hypoCOFs according to their adsorption selectivities and identified the top-performing materials with the highest selectivities for every separation except O_2_/N_2_ (for which none of the materials is strongly selective). From these results, we selected three materials that consistently appeared among the top ten candidates for at least two different gas separations.

The triazine-based materials, hCOF-268193 and hCOF-268595, were identified as top materials for CO_2_-based separations, while hCOF-268547 was identified as a top material for CH_4_-based separations. Structural representations, features, and molecular fingerprints that we extracted for these ReDD-hypoCOFs are given in Figure 9. hCOF-268193 was identified to have the highest CO_2_/H_2_ selectivity (189.5) and it was ranked among the top ten materials for CH_4_/H_2_ separation as well, with a selectivity of 42.8. The fingerprint analysis of this material shows that it consists of nitrogen enriched rings, and aromatic rings (bit-78, bit-121). Additionally, it has fluorinated linkers observed as bit-107. These fluorine groups, together with the existence of triazine subunits and narrow-pored structure, favor the adsorption of CO_2_,^48,49^ resulting in high CO_2_ uptake (3.7 mol/kg) and selectivity. hCOF-268595 was identified as a top material for CO_2_/CH_4_ and CO_2_/N_2_ separations, as it exhibits the highest CO_2_/CH_4_ selectivity of 9.2 and a high CO_2_/N_2_ selectivity of 20. It consists of N-enriched aromatic rings and fluorinated linkers, enhancing the electrostatic interactions with CO_2_ and favoring its adsorption over CH_4_ and N_2_.^50^ This results in a high CO_2_ uptake (5.9 mol/kg), and also high CO_2_ selectivities.

**Figure 9:**
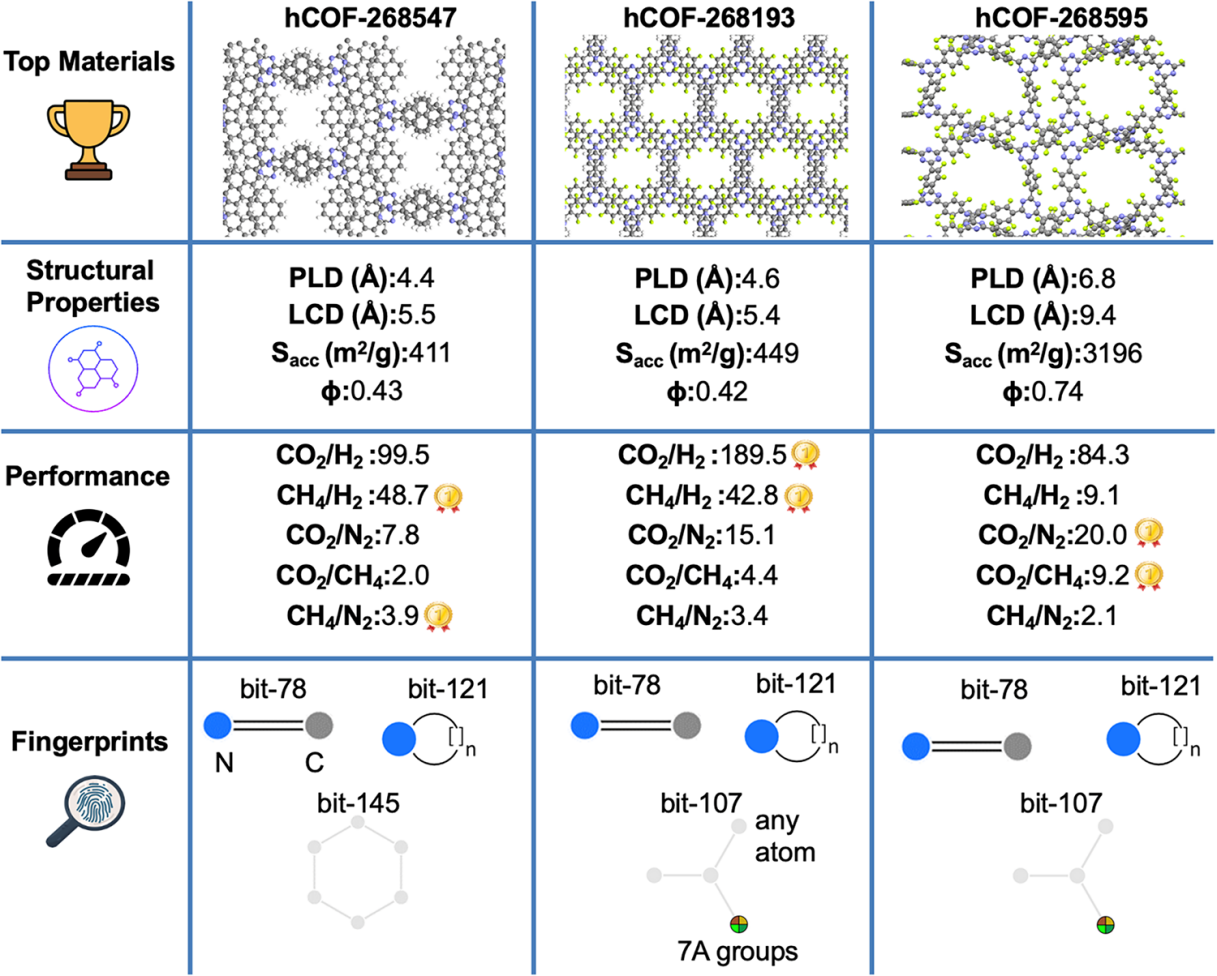
Detailed chemical analysis of three triazine-based ReDD-hypoCOFs having the highest selectivities for gas separations. Gray, blue, white, and green atoms signify carbon, nitrogen, hydrogen and fluorine atoms in the structures, respectively.

We observed that the structures with narrow pores (<10 Å) and low porosities (<0.7) exhibit high CH_4_/H_2_ separation performance. hCOF-268547 achieved the highest CH_4_/H_2_ selectivity (48.7) among our material space of interest. The fingerprint analysis shows that it only consists of light elements (carbon, nitrogen, and hydrogen), which are linked in 5-element, or 6-element constituted ring structures (bit-78, bit-121, and bit-145), thus there is no specific functional group favoring CH_4_ adsorption. We inferred that its high selectivity arises from its structural properties, as the narrow pores preferentially confine CH_4_ over H_2_. Overall, we concluded that structural properties, such as pore size and porosity, determine the CH_4_/H_2_ separation performance of ReDD-hypoCOFs, while the existence of CO_2_-favoring N-enriched aromatic building blocks, particularly triazine units, and fluorinated or electron-deficient linkers enhance CO_2_/H_2_, CO_2_/CH_4_, and CO_2_/N_2_ separation performance of ReDD-hypoCOFs. Importantly, these features are not specific to a single structure, but present in distinct ReDD-hypoCOFs as well.

To directly test whether the structural and chemical properties identified for the top-performing ReDD-hypoCOFs (Figure 9) extend to other materials in the database, we performed a reverse search over the ReDD-hypoCOF set. We focused on triazine family and identified the 50 materials showing the highest selectivities for each gas separation. 87 and 81 unique structures were found for CO_2_-based (CO_2_/CH_4_, CO_2_/N_2_, and CO_2_/H_2_) and CH_4_-based (CH_4_/H_2_ and CH_4_/N_2_) separations, respectively. These materials have narrow pores, mostly between 5 and 9 Å and porosities in the range of 0.4–0.7. Nearly all the structures with the highest CO_2_/CH_4_, CO_2_/N_2_, and CO_2_/H_2_ selectivities contain halogens, whereas only half of the structures with the highest CH_4_/H_2_ and CH_4_/N_2_ selectivities have halogens. For instance, the top-performing structure, hCOF-ReDD-268193, has the highest CO_2_/H_2_ selectivity of 189.5, while the remaining structures with high CO_2_/H_2_ selectivities exhibit an average of 125.4, indicating that several materials with similar structural and chemical properties to the most promising candidate exist. Here, it is important to emphasize that the top-performing materials that we discussed above were not identified for a single separation, but instead consistently exhibit strong performance across multiple separations.

ML models can be easily transferred to different COF databases by tuning model parameters. Maintaining an appropriate balance between training and test sets is important to prevent overfitting and ensure reliable generalization. Importantly, the relative contribution of descriptors can differ significantly from one COF database to another, depending on the underlying chemical diversity and structural features of the materials. While previously developed ML models can be partially transferred to some linker families, others require retraining to accurately capture their structure–property relationships. The large and chemically diverse ReDD-COFFEE database offers a key advantage over smaller experimental databases because it enables the exploration of underrepresented yet experimentally relevant linkage types and wider pore sizes. This expanded material space allows the identification of promising COFs that would remain inaccessible in smaller data sets and significantly improves the robustness and interpretability of ML models.

The synthesizability of computationally generated materials is also important to utilize the hypothetical structures in real applications. As a result of the algorithm they developed to generate hypothetical COFs, Smit’s group obtained COF-300 and TAPB-PDA COF structures, compared the XRD patterns of the structures generated by their algorithm with those of the existing experimental structures, and showed that their algorithm is capable of producing synthesizable structures.^51^ Based on this and considering the ongoing advances in COF synthesis and crystallization control, we expect that some of the promising ReDD-hypoCOFs can be experimentally synthesized in the near future.^52^

## 4 Conclusion

This work examined gas adsorption and separation potentials of acylhydrazone, azine, and triazine families present in the ReDD-COFFEE database using molecular simulations and ML models. We assessed CO_2_/CH_4_, CO_2_/H_2_, CO_2_/N_2_, CH_4_/H_2_, CH_4_/N_2_ and O_2_/N_2_ selectivities of 24092 different types of materials and showed that triazine-based ReDD-hypoCOFs are a very promising family that outperform acylhydrazone and azine families for all three CO_2_ separations and CH_4_/H_2_ separation. Compared to synthesized COFs (CoRE COFs) and previously reported hypoCOFs, ReDD-hypoCOFs exhibit higher selectivities for CO_2_/H_2_ separation, while achieving comparable selectivities in other gas separations. Detailed chemical analyses of the triazine-based COFs with the highest CO_2_ selectivities revealed that electron-deficient aromatic units, nitrogen-containing basic sites, and narrow pore environments collectively enhance CO_2_ affinity, thereby leading to superior separation performances. Overall, considering the strong potential of ReDD-hypoCOFs for various gas separation applications, future efforts for synthesis and testing would be critical.

## Supplementary Material



## Notes

Distributions of computed structural features of all three families of ReDD-hypoCOFs; the box plot data of structural and energetic comparisons of subsets of acylhydrazone and azine ReDD-hypoCOFs; correlation matrix for gas uptakes and structural, chemical, energetic descriptors; the comparison of simulated and ML-predicted uptakes for unseen triazine ReDD-hypoCOFs; the relationships between simulated and ML-predicted CO_2_ and CH_4_ uptakes and their Henry’s constants for CoRE COFs, hypoCOFs and ReDD-hypoCOFs; the SHAP analysis of new ML models trained with the simulation data of triazine ReDD-hypoCOFs; the number of total, filtered, and simulated structures across different ReDD-hypoCOF subsets, the statistical accuracy metrics calculated to evaluate ML models; ML pipelines and their parameters based on the target gas adsorption properties of triazine-based ReDD-hypoCOFs (PDF)
