# Supplementary material for: ReDD-COFFEE under the Lens: Revealing Adsorption and Separation Performances of Hypothetical COFs Using Molecular Simulations and Machine Learning
Source: Ind Eng Chem Res. 2026 Feb 15;65(7):3920–31. doi: 10.1021/acs.iecr.5c04806 (PMC12947673; doi:10.1021/acs.iecr.5c04806)
Supplement: Supplementary file 1 [file ie5c04806_si_001.pdf]

Supporting Information

ReDD-COFFEE Under the Lens: Revealing Adsorption and Separation Performances of Hypothetical COFs Using Molecular Simulations and Machine Learning

Hilal Ozyurt, Gokhan Onder Aksu, Hasan Can Gulbalkan, and Seda Keskin*

Department of Chemical and Biological Engineering, Koc University, Rumelifeneri Yolu, Sariyer, 34450, Istanbul, Turkey

Submitted to *Industrial & Engineering Chemistry Research*

*Corresponding author. E-mail: skeskin@ku.edu.tr Phone: +90 (212) 338-1362.

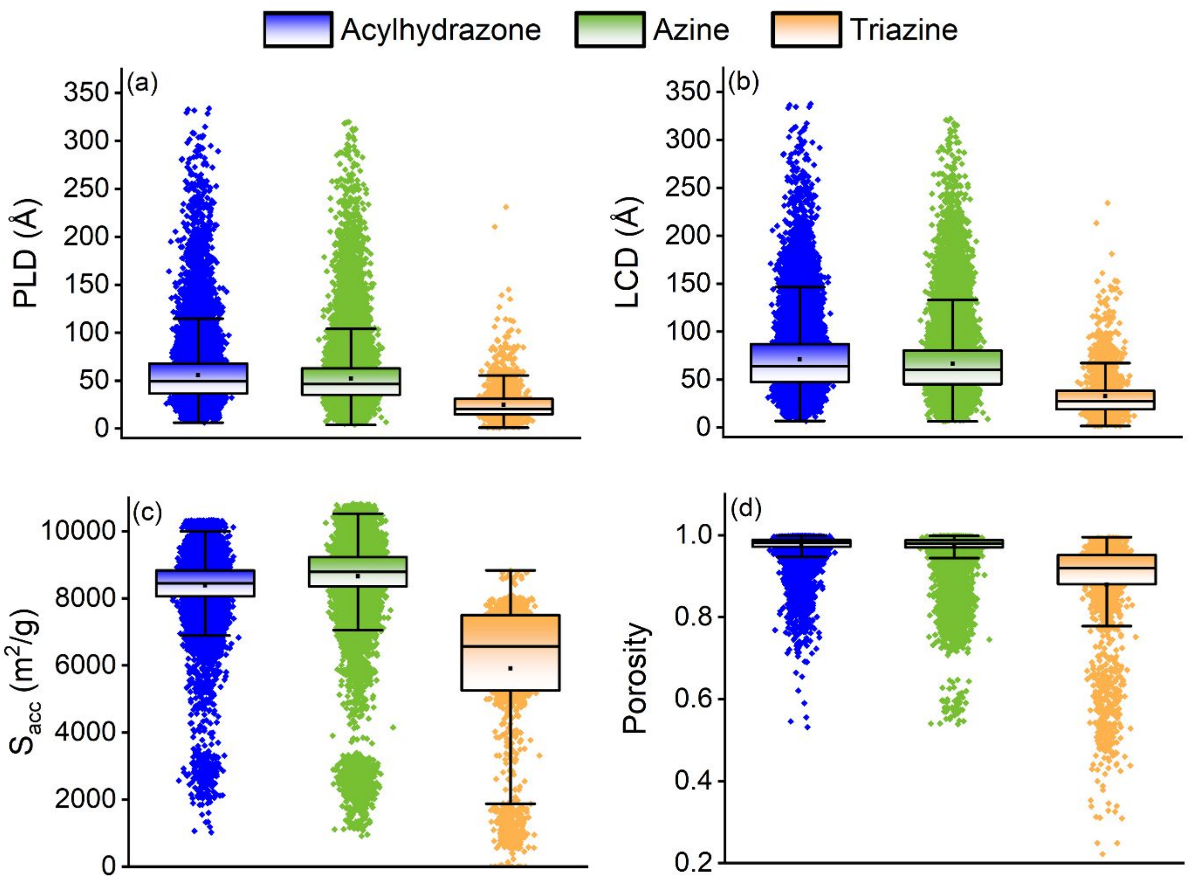

Figure S1. Computed structural features; (a) PLD, (b) LCD, (c) surface area, and (d) porosity of all 39412 acylhydrazone, 55308 azine, and 2641 triazine ReDD-hypoCOFs, respectively.

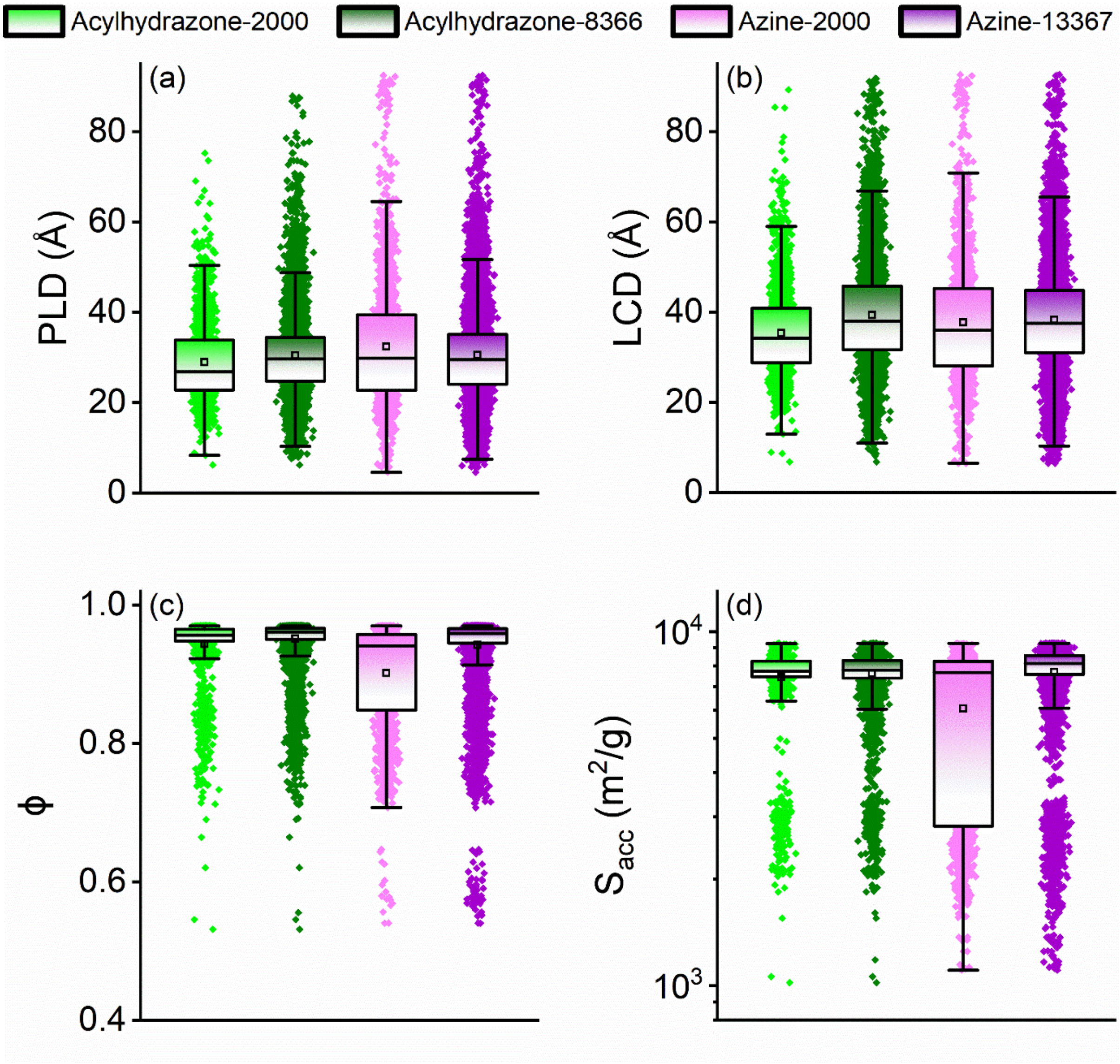

Figure S2. The comparison of subsets of acylhydrazone and azine ReDD-hypoCOFs with its refined material space with respect to (a) PLD, (b) LCD, (c) porosity and (d) surface area, respectively.

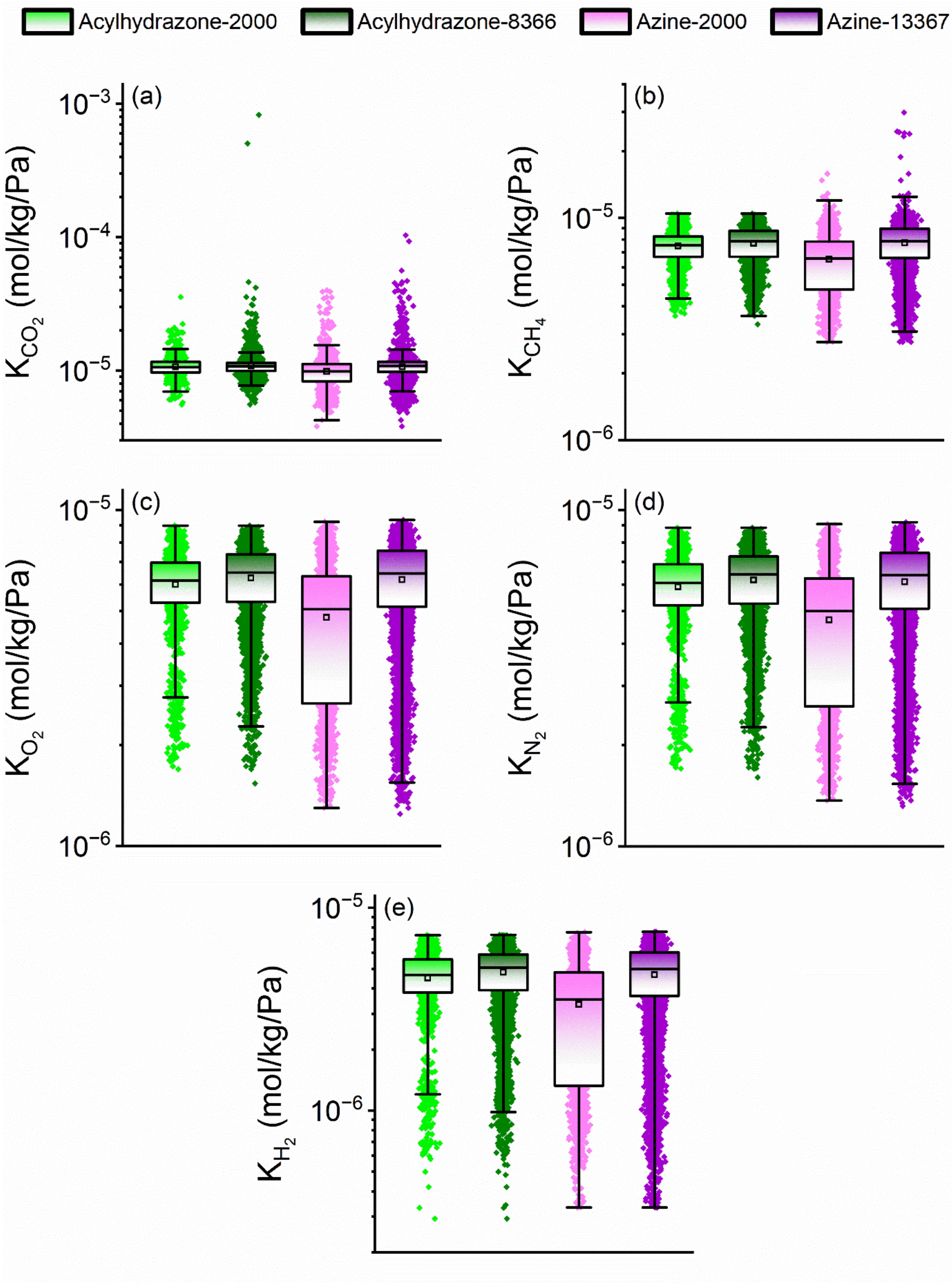

Figure S3. The comparison of subsets of acylhydrazone and azine ReDD-hypoCOFs with its refined material space with respect to their Henry's constants of (a) CO_2, (b) CH_4, (c) O_2, (d) N_2, and (e) H_2, respectively.

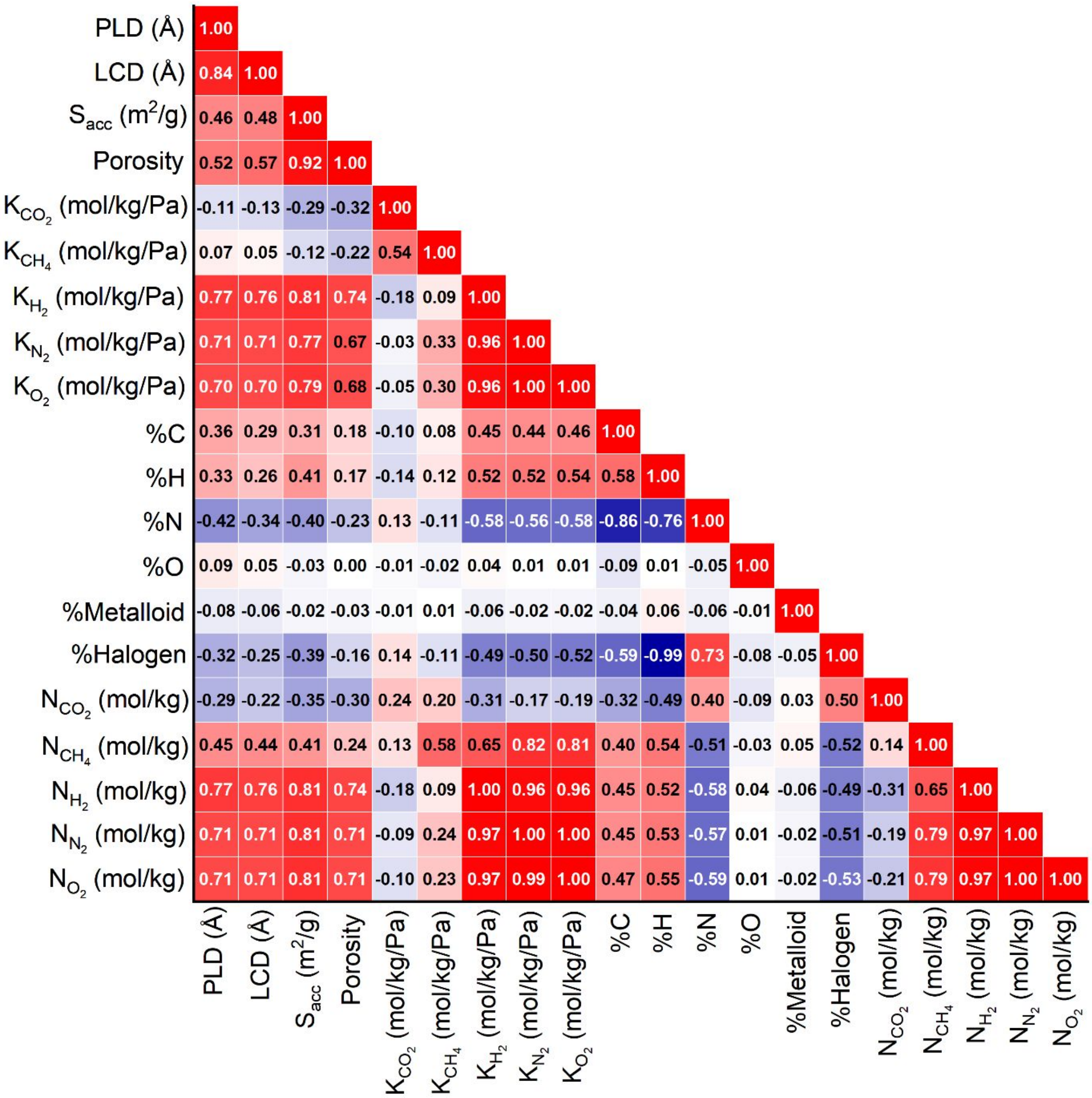

Figure S4. Correlation matrix for gas uptakes, and structural, chemical, energetic descriptors of 2359 triazine-based ReDD-hypoCOFs. Pearson coefficients (r) are provided for the relationship between each descriptor.

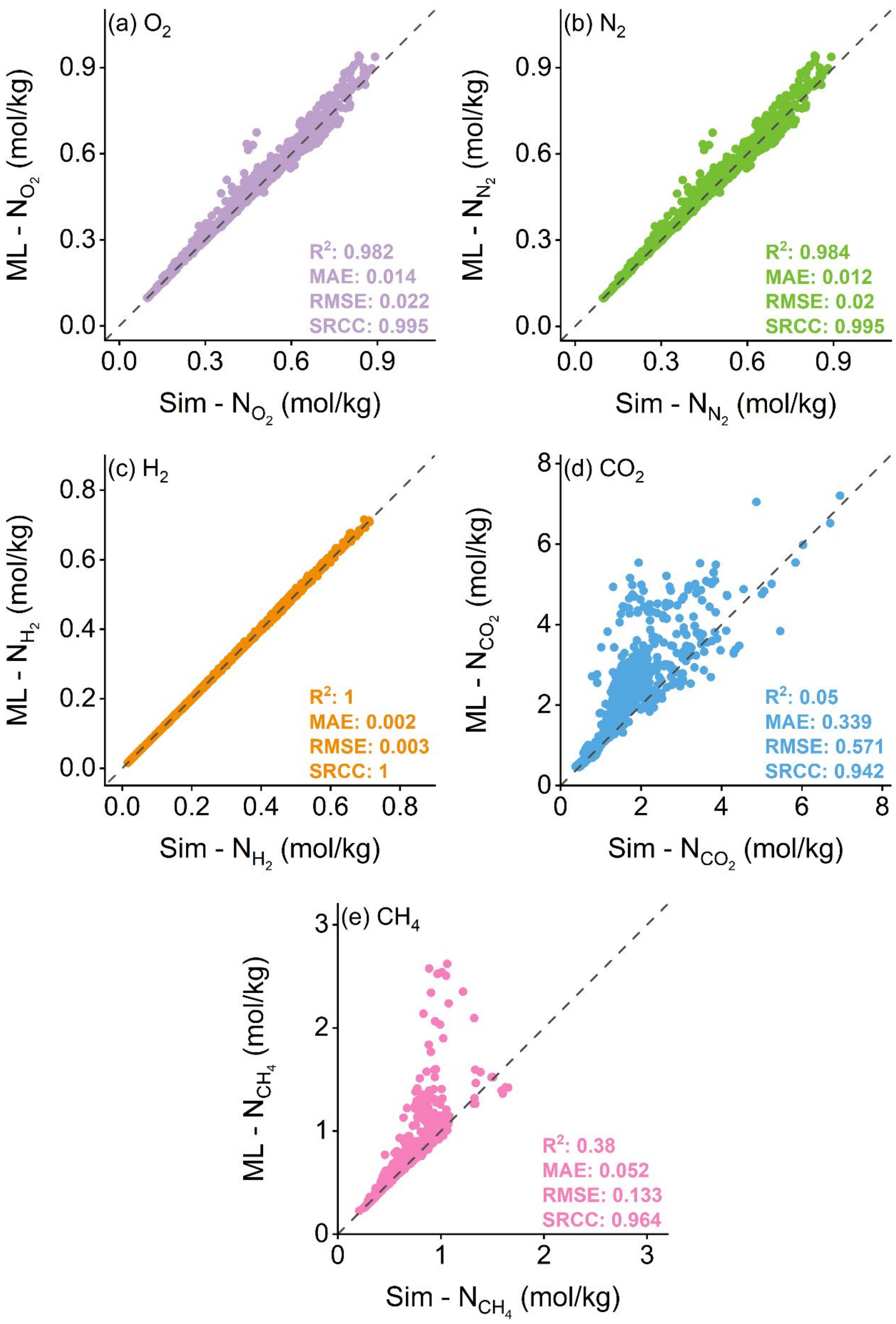

Figure S5. The comparison of simulated and ML-predicted (original model) (a) O_2, (b) N_2, (c) H_2, (d) CO_2, and (e) CH_4 uptakes for 2359 unseen triazine ReDD-hypoCOFs for at 1 bar and 298 K.

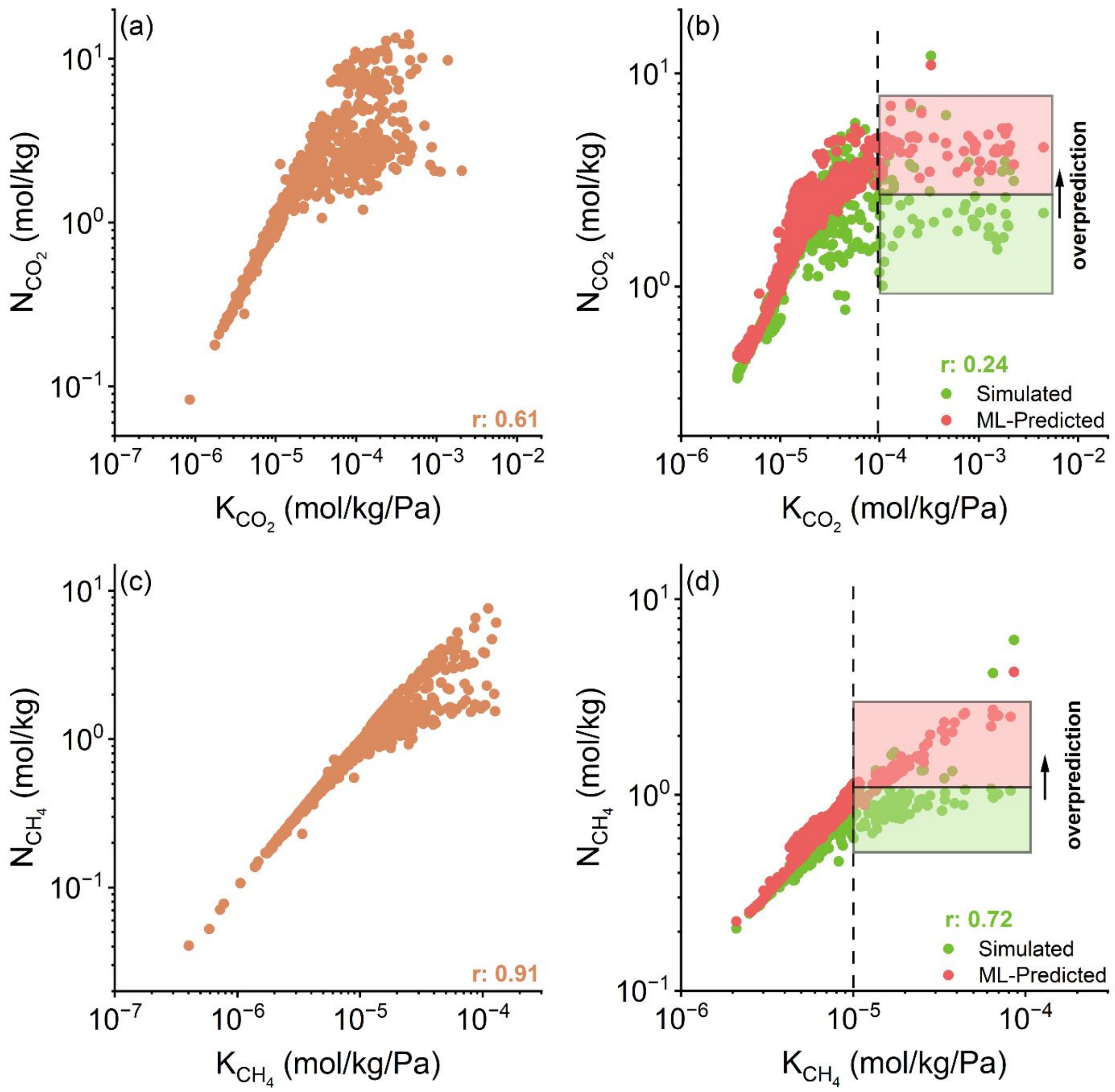

Figure S6. (a) The relationship between simulated CO_2 uptakes of 1060 CoRE COFs and 563 Smit's hypoCOFs (in the training set of our original ML models) and Henry's constants. (b) The relationship between simulated and ML-predicted CO_2 uptakes of 2359 triazine ReDD-hypoCOFs and Henry's constants. (c) The relationship between simulated CH_4 uptakes of 1060 CoRE COFs and 563 Smit's hypoCOFs (in the training set of our original ML models) and Henry's constants. (d) The relationship between simulated and ML-predicted CH_4 uptakes of 2359 triazine ReDD-hypoCOFs and their Henry's constants of CH_4. Pearson's constants (r) between simulated uptakes and Henry's constants were given.

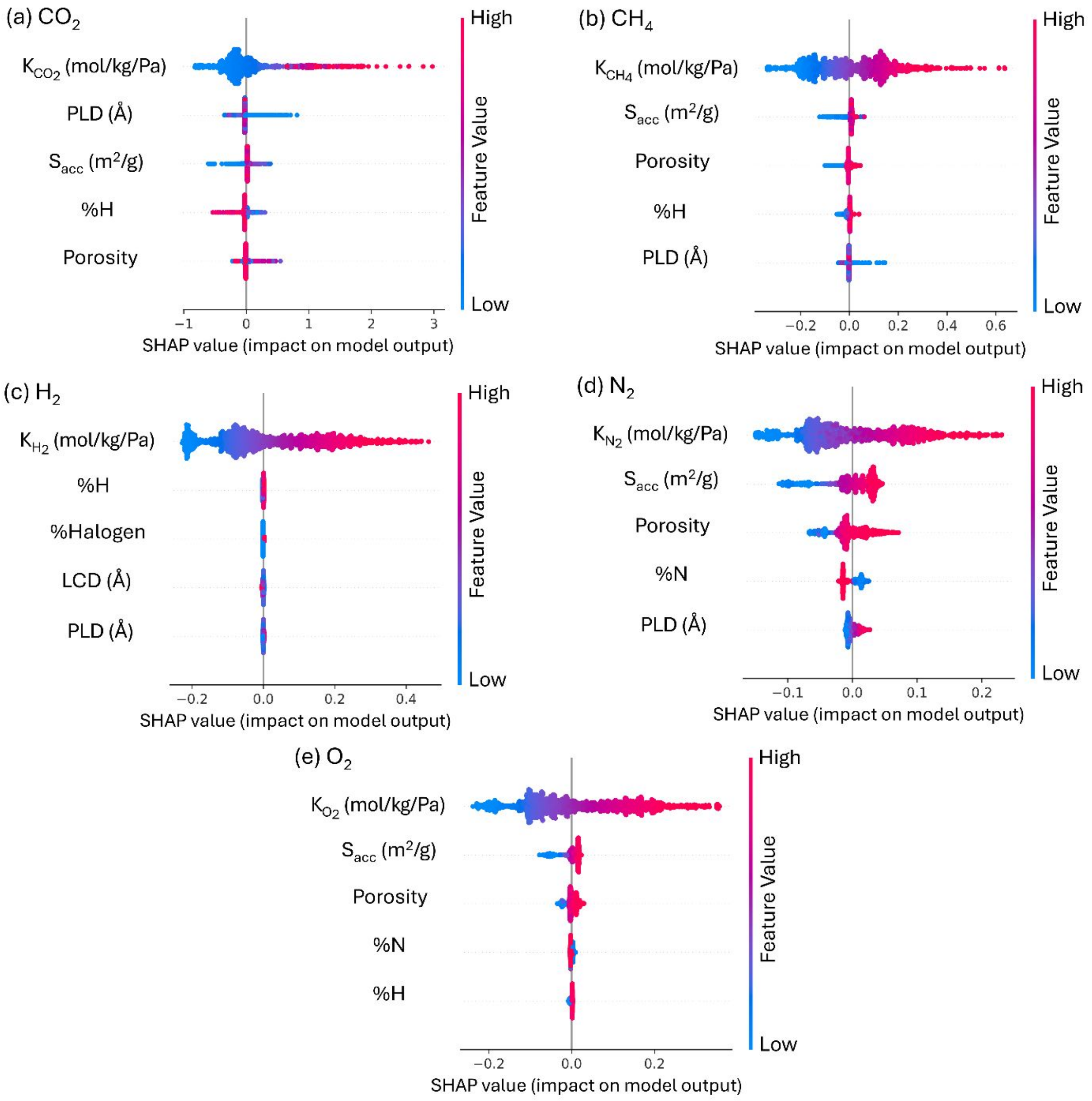

Figure S7. SHAP feature importance distributions for the most important five features of new ML models trained with the simulation data of triazine ReDD-hypoCOFs, which were developed to predict (a) CO_2, (b) CH_4, (c) H_2, (d) N_2 and (e) O_2 gas uptakes of 2359 ReDD-hypoCOFs, respectively.

Table S1. Number of total, filtered, and simulated structures across different ReDD-hypoCOF subsets.

ReDD-hypoCOF Subset	Total Structures	Refined Structures	Simulated Structures
**Acylhydrazone**	39412	8366	2000
**Azine**	55306	13367	2000
**Triazine**	2641	2359	2359

Table S2. The statistical accuracy metrics calculated to evaluate ML models.

	**Formula**
**Pearson Correlation Constant (r)**	$\frac{\sum_{i=1}^{n}(x_i-\bar{x})(y_i-\bar{y})}{\sqrt{\sum_{i=1}^{n}(x_i-\bar{x})^2}\sqrt{\sum_{i=1}^{n}(y_i-\bar{y})^2}}$
**Coefficient of Determination (R^2)**	$1-\frac{1}{M}\sum_{m=1}^{M}(\bar{y}-\hat{y})^2 / \frac{1}{M}\sum_{m=1}^{M}(y-\hat{y})^2$
**Mean Absolute Error (MAE)**	$\sum_{m=1}^{M}\lvert y-\hat{y}\rvert/M$
**Root Mean Square Error (RMSE)**	$\sqrt{\sum_{m=1}^{M}(y-\hat{y})^2/M}$
**Spearman Ranking Correlation Coefficient (SRCC)**	$1-\frac{6\sum d_i^2}{M(M^2-1)}$

M: the number of samples, x_i: i^{th} data point of variable 1, y_i: i^{th} data point of variable 2, y: simulated value, $\hat{y}$: predicted value, $\bar{y}$: average of the simulated value, d_i: the difference between the ranks of corresponding variables.

Table S3. The ML pipelines and their parameters based on each target gas adsorption properties of 2359 triazine-based ReDD-hypoCOFs at 1 bar, 298 K.

Property	Best Pipeline with Parameters
CO_2	XGBRegressor(input_matrix, learning_rate=0.1, max_depth=8, min_child_weight=9, n_estimators=100, n_jobs=1, objective=reg:squarederror, subsample=0.9000000000000001, verbosity=0)
CH_4	XGBRegressor(input_matrix, learning_rate=0.1, max_depth=9, min_child_weight=6, n_estimators=100, n_jobs=1, objective=reg:squarederror, subsample=0.6500000000000001, verbosity=0)
H_2	RidgeCV(MaxAbsScaler(input_matrix))
N_2	RandomForestRegressor(ExtraTreesRegressor(MinMaxScaler(input_matrix), bootstrap=False, max_features=0.8500000000000001, min_samples_leaf=14, min_samples_split=11, n_estimators=100), bootstrap=True, max_features=0.8500000000000001, min_samples_leaf=3, min_samples_split=3, n_estimators=100)
O_2	ExtraTreesRegressor(input_matrix, bootstrap=False, max_features=0.8, min_samples_leaf=2, min_samples_split=3, n_estimators=100)
